# Molecular Dynamics Simulations of Ion Permeation in Human Voltage-Gated Sodium Channels

**DOI:** 10.1021/acs.jctc.2c00990

**Published:** 2023-04-28

**Authors:** Giulio Alberini, Sergio Alexis Paz, Beatrice Corradi, Cameron F. Abrams, Fabio Benfenati, Luca Maragliano

**Affiliations:** † Center for Synaptic Neuroscience and Technology (NSYN@UniGe), Istituto Italiano di Tecnologia, Largo Rosanna Benzi 10, 16132 Genova, Italy; ‡ IRCCS Ospedale Policlinico San Martino, Largo Rosanna Benzi 10, 16132 Genova, Italy; § Departamento de Química Teórica y Computacional, Facultad de Ciencias Químicas, Universidad Nacional de Córdoba, X5000HUA Córdoba, Argentina; ∥ Consejo Nacional de Investigaciones Científicas y Técnicas (CONICET), Instituto de Fisicoquímica de Córdoba (INFIQC), X5000HUA Córdoba, Argentina; ⊥ Department of Experimental Medicine, Università degli Studi di Genova, Viale Benedetto XV 3, 16132 Genova, Italy; ∇ Department of Chemical and Biological Engineering, Drexel University, Philadelphia, Pennsylvania 19104, United States; ○ Department of Life and Environmental Sciences, Polytechnic University of Marche, Via Brecce Bianche, 60131 Ancona, Italy

## Abstract

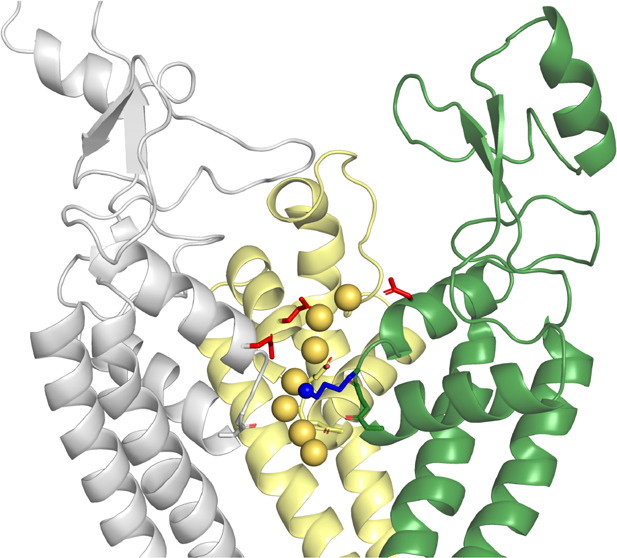

The recent determination of cryo-EM structures of voltage-gated sodium (Na_v_) channels has revealed many details of these proteins. However, knowledge of ionic permeation through the Na_v_ pore remains limited. In this work, we performed atomistic molecular dynamics (MD) simulations to study the structural features of various neuronal Na_v_ channels based on homology modeling of the cryo-EM structure of the human Na_v_1.4 channel and, in addition, on the recently resolved configuration for Na_v_1.2. In particular, single Na^+^ permeation events during standard MD runs suggest that the ion resides in the inner part of the Na_v_ selectivity filter (SF). On-the-fly free energy parametrization (OTFP) temperature-accelerated molecular dynamics (TAMD) was also used to calculate two-dimensional free energy surfaces (FESs) related to single/double Na^+^ translocation through the SF of the homology-based Na_v_1.2 model and the cryo-EM Na_v_1.2 structure, with different realizations of the DEKA filter domain. These additional simulations revealed distinct mechanisms for single and double Na^+^ permeation through the wild-type SF, which has a charged lysine in the DEKA ring. Moreover, the configurations of the ions in the SF corresponding to the metastable states of the FESs are specific for each SF motif. Overall, the description of these mechanisms gives us new insights into ion conduction in human Na_v_ cryo-EM-based and cryo-EM configurations that could advance understanding of these systems and how they differ from potassium and bacterial Na_v_ channels.

## Introduction

Voltage-gated sodium (Na_v_) channels are integral proteins that are responsible for Na^+^ conduction across the membrane of various excitable cells, where they participate in essential functions including the triggering of neuronal action potential.^1−7^ Several Na_v_ mutations induce dysfunctions linked to a variety of pathological conditions such as epileptic seizures and motor neuron disease and pain,^8−13^ making them a major target for the design of efficient therapeutic strategies.^14−19^ Despite their physiological importance, little is known on their structural mechanisms, also because of the limited availability of human Na_v_ structures. The Na_v_ channels family includes both prokaryotic (bacterial) and eukaryotic members, which are evolutionarily divergent even if they share the same main functions.^20,21^ In particular, the eukaryotic subfamily of human Na_v_ proteins comprises nine homologues, named Na_v_1.1 to Na_v_1.9, all characterized by highly conserved sequences.^22−24^ These channels show tissue-specific distribution, with Na_v_1.1, Na_v_1.2, and Na_v_1.6 expressed in the central nervous system (CNS).^15^ Moreover, unlike bacterial Na_v_ channels, which are made up of four separated subunits, the human Na_v_ channels are formed by four different domains, also called repeats (indicated as Rep. DI–DIV), connected together to form a single long polypeptide chain known as the ion-conducting α subunit. When viewed from the extracellular side, the DI–DIV domains are assembled in a clockwise fashion, each containing six transmembrane (TM) α helices, named S1 to S6. The S1–S4 segments of each repeat form four independent voltage-sensing domains (VSDs),^5,25−27^ while the last two helices, S5 and S6, are assembled to generate the pore domain (PD).^28−38^ Between the S5 and S6 segments there is a motif (named P-loop) that includes the Na^+^ selectivity filter (SF),^15,39^ which ensures the selective permeation of Na^+^ ions and differs between bacterial and eukaryotic channels. In particular, bacterial Na_v_ channels display a ring of four E side chains (EEEE),^39^ as evidenced by high-resolution crystallographic structures.^40−44^

Several independent studies used molecular dynamics (MD) simulations to investigate the structural properties of bacterial Na_v_ proteins.^45^ According to refs (34) and (46), the main results achieved could be summarized as follows:

- A first group of simulations revealed information regarding the binding sites in the SF and their multi-ion occupancy.^47−60^
- Additional microsecond-time-scale MD simulations showed that the SF symmetric configuration of the crystal structures can be broken, affecting both the permeation and the selectivity mechanisms.^53,58,59,61,62^

The number of Na^+^ ions that permeate the SF is also a central issue. The observation of a multi-ion process is in agreement with the recent crystallographic structures of a bacterial Na_v_Ms channel introduced in ref (44). Moreover, the X-ray crystal structure^42^ of the closed conformation of Na_v_Ae1p, another prokaryotic Na_v_ channel, is characterized by an outer ion site in the SF that suggests multiple ion-binding sites.^15^

Mammalian Na_v_ channels replace the EEEE motif with a D_I_E_II_K_III_A_IV_ ring^39,63−65^ (here, following the notation in ref (46), subscripts indicate the Na_v_ domain associated with each residue) that includes a lysine side chain, which selectively slows the transport of K^+^ over Na^+^.^66−69^ The SF is formed by the four side chains of the D_I_E_II_K_III_A_IV_ signature at the extracellular side and, at the inner side, by the couple of backbone carbonyl oxygen atoms belonging to the two preceding residues in each repeat.^63−65^ Indeed, since they are not involved in hydrogen bonds with other residues, these two inner rings of carbonyls are free to interact with the permeating cations.^63−65,70^ In addition, both experimental results and Monte Carlo simulation studies suggested the pivotal role of the E and K residues in the DEKA ring.^66,70^ Several investigations demonstrated that the specific position of the residues in the SF is essential to preserve the correct selectivity.^66,71^ Of particular interest, the DEEA variant^22,72−77^ reverts the SF to an ancestral Ca^2+^-selective configuration. In the case of the Na_v_1.2 channel, the alteration of the third residue in the DEKA motif (K1422E) affects the organism with both loss-of-function and gain-of-function effects.^67,71,76^

In addition to the DEKA signature, human channels (with the exception of Na_v_1.7) display a well-conserved^65,78^ outer ring consisting of four charged residues, E_I_E_II_D_III_D_IV_ (again, subscripts indicate the Na_v_ domain associated with each residue^46^), which is supposed to increase the electrostatic attraction and conductance of extracellular cations but not selectivity.^71,78^ Remarkably, the residues in both the EEDD and DEKA motifs are asymmetric in their position along the pore axis and characterized by a high degree of flexibility.^65^ To cope with such structural complexity, in the following we will refer to the region including the conductive EEDD signature, the DEKA motif, and the two inner rings as the conductivity/selectivity filter (C/SF). As for ionic occupancy, although early experiments suggested that Na_v_ channels are characterized by multiple binding sites,^79−82^ simultaneous binding of multiple ions is not universally accepted, leading to disagreements on the mechanism of ionic conduction.^79−84^

Previous computational studies examined also the role of the lysine belonging to the DEKA ring in conduction and selectivity, but they were based on models using modified bacterial structures^46,85,86^ or homology models of mammalian Na_v_ channels from bacterial templates.^87−90^ In two separate articles,^85,86^ the authors replaced the ring in the bacterial Na_v_Rh channel with the DEKA motif and performed MD simulations, describing the screening process of the K residue, which pushes the Na^+^ ion toward the D/E residues. On the other hand, the MD studies of refs (87−90), based on a homology model of the rat Na_v_1.4 pore-only channel from the bacterial Na_v_Ab structure, showed an essential displacement of the lysine in order to allow proper ionic permeation. More recently, the results based on extended MD simulations in ref (46) suggest that the role of lysine in conduction and selectivity is strongly dependent on its protonation state. When the K residue is deprotonated (i.e., uncharged), two- and three-ion occupancies are preferred. On the contrary, when the residue is protonated (positively charged), a reduced occupancy (one or two) is observed.

Progress in understanding the molecular details of Na_v_ channels was strongly accelerated by the recent publication of various cryo-EM structures of eukaryotic Na^+^ channels (for an updated summary, see ref (91)). However, the use of these structures in MD simulations remains limited and debated,^34,46,91^ mostly because their global atomic resolution is considered too low. In particular, the first two eukaryotic (nonmammalian) channels obtained, Na_v_PaS (PDB ID 5X0M, ref (63)) and EeNa_v_1.4 (PDB ID 5XSY, ref (64)), have a resolution of 3.8 and 4.0 Å, respectively, and many side chains belonging to the highly flexible C/SF are not resolved, hindering the investigation of processes such as ion permeation, selectivity, and drug binding. The most recent publication of the first human Na_v_ channel (Na_v_1.4-β1 complex), with a global resolution of 3.2 Å and a local resolution up to 2.8 Å in the SF, represents a significant improvement in terms of quality of experimental Na_v_ structures. However, also in this case, the use in MD simulations requires caution, since the resolution is highly variable in different parts of the protein. This notwithstanding, the growing population of cryo-EM Na_v_ structures constitutes an important platform to study their structural and functional properties, and the number of MD-based studies is constantly growing. The exploration of ionic permeation, however, remains mostly focused on bacterial structures and derived models, and the transferability of results to eukaryotic species^63−65^ has been investigated only in a few works,^70,91−93^ so that a detailed thermodynamic characterization of the process in mammalian channels is still missing.

In this work, we aim to investigate the residence of Na^+^ ions in structures of neuronal Na^+^ channels by means of MD simulations and free energy (FE) calculations. To this aim, we used both high-quality homology-based Na_v_ models from cryo-EM templates and experimental cryo-EM Na_v_ structures. The application of homology modeling is justified by the high sequence identity among human Na_v_ (hNa_v_) channels that spans from 85% to 91% in the TM region, thus ensuring a remarkable standard of the output configurations from the comparative modeling.^94^ In particular, starting from the first cryo-EM structure of a human Na_v_ channel (Na_v_1.4, PDB ID 6AGF,^65^ at 3.20 Å resolution), we obtained the homology-modeled structures of the α subunits of three neural Na_v_ channels (Na_v_1.1, Na_v_1.2, and Na_v_1.6). We performed all-atom unbiased MD simulations to assess the structural stability of these structures in the wild type (WT) configuration immersed in a water-membrane environment at a 150 mM physiological concentration of NaCl. Na^+^ permeation events from the external space to the internal cavity were observed in all the systems. Then, focusing on the Na_v_1.2 model, as a paradigmatic example of neurogical Na_v_s, we used pore-only configurations (i.e., without the VSDs) to study the Na^+^ permeation in the C/SF via FE surface (FES) calculations using the temperature-accelerated MD/on-the-fly free energy parametrization (TAMD/OTFP) method.^95−98^ Special attention was devoted to the role of SF motif in the WT and DEEA structure, considering both the charged and uncharged version of the K or E1422 residue. The investigation was replicated on the more recent cryo-EM Na_v_1.2 structure (PDB ID 6J8E,^99^ at 3.00 Å resolution, based on the aforementioned Na_v_1.4 structure). The overall conformation of the experimental Na_v_1.2 is substantially identical to the previous Na_v_1.4 template with a root-mean-square deviation (RMSD) of 0.696 Å over 981 Cα atoms, according to the analysis exposed in ref (99). Based on this observation, not surprisingly, the results of the MD simulations performed with the latter structure nicely fit with those obtained with the starting homology-based model. Our simulations describe distinct mechanisms for single and double Na^+^ ions during permeation through the WT filter, with the charged K1422 residue. Remarkably, in the case of the WT configuration, both standard MD runs and FE calculations highlight single Na^+^ permeation events, in the course of which the ion stays in a global FE minimum in the inner part of the Na_v_ SF. These ionic configurations observed for the WT with the charged K1422 residue are specific of the WT DEKA and differ from those obtained for the other filter motifs. Our results provide a first attempt to describe the permeation mechanisms of Na^+^ translocation through neuronal human Na_v_ channels using the information provided by the recent cryo-EM hNa_v_ structures. Globally, these outcomes could help in describing the complex mechanism of ion permeation of these systems.

## Materials and Methods

This section is organized in two subsections. In the first one, we report the information related to the structural modeling and the standard MD simulations, including all the details of the analysis performed. In the second subsection, we introduce the details of the OTFP/TAMD simulations. A brief descrition of the method^96−98,100^ is also included.

### Structural Modeling and Standard MD Simulations

#### Structural Modeling: Template Choice

When we started this project, no experimental structure was available for mammalian neuronal Na_v_1.1, -1.2, and -1.6 channels. Therefore, we first relied on homology-based modeling to build accurate structural models for these proteins. The cryo-EM structure of the human Na_v_1.4 α protein, in complex with the auxiliary β1 subunit, introduced in ref (65) (PDB ID 6AGF), has been used as template. This structure is characterized by a detailed description of the pore domain, including the C/SF that regulates the permeation of ions. In particular, the critical DEKA motif is located in the inner part of the cavity and was reliably resolved with a local resolution of up to ∼2.8 Å. This value is comparable to that of other membrane protein structures employed in MD simulations. As an example, the inactive crystal structure of the μ-opioid receptor (MOR), resolved at 2.8 Å (PDB ID 4DKL(101)), was used to investigate computationally ion translocation through the allosteric sites.^102−104^ In Table 1, we introduce the sequence of the different domains modeled for each channel. The sequence identity between neuronal hNa_v_ channels and the hNa_v_1.4 homologue spans from ∼85% to ∼91% in the TM region. Such values are generally considered appropriate to obtain reliable homology-based structures at the atomic resolution (see for example ref (94)). As an example, a similar criterion (∼84% identity in the TM pore region) was used in ref (105) to model the hNa_v_1.5 channel from the cryo-EM structure of the electric eel Na_v_1.4 channel in order to study atomistic interactions of antiarrhythmic and local anesthetic drugs with the protein itself. Our homology-based structures include the four Rep. DI–DIV TM domains, plus the intracellular link between DIII and DIV (III–IV link). The long extracellular loop located in the DI part was not included in the modeling because it is unresolved in the template. Therefore, the residues adjacent to the missing region were connected, forming one single DI polypeptide chain.

**Table 1:** List of All the Residues Included in Homology Modeling; The Starting UNIPROT IDs (UIDs) with the Associated Human Sequences Are Included

Channel	Na_v_1.1 (UID: P35498)	Na_v_1.2 (UID: Q99250)	Na_v_1.6 (UID: Q9UQD0)
Rep. DI	L129–A420 (no A285-I305)	I125–A427 (no N285–T305)	V133–A408 (no E290–G299)
Rep. DII	F769–L991	V755–L983	F754–L976
Rep. DIII	F1220–I1479	F1210–I1473	F1200–F1459
III–IV Link	G1480–V1542	D1474–D1526	I1460–A1523
Rep. DIV	F1543– I1785	F1527–I1775	F1524–I1765

#### Notation of the SF Configurations

Here we report the notation used to differentiate the Na_v_ SF configurations investigated in this work:

- **DEK**^+1^**A**, WT configuration of the DEKA ring with the positively charged lysine.
- **DEK**^0^**A**, WT configuration of the DEKA ring with uncharged lysine.
- **DEE**^–1^**A**, mutated configuration of the DEKA ring with the charged glutamate in place of the lysine.
- **DEE**^0^**A**, mutated configuration of the DEKA ring with uncharged glutamate in place of the lysine.

#### Structural Modeling of Neuronal Na_v_ Channels

**Figure 1:**
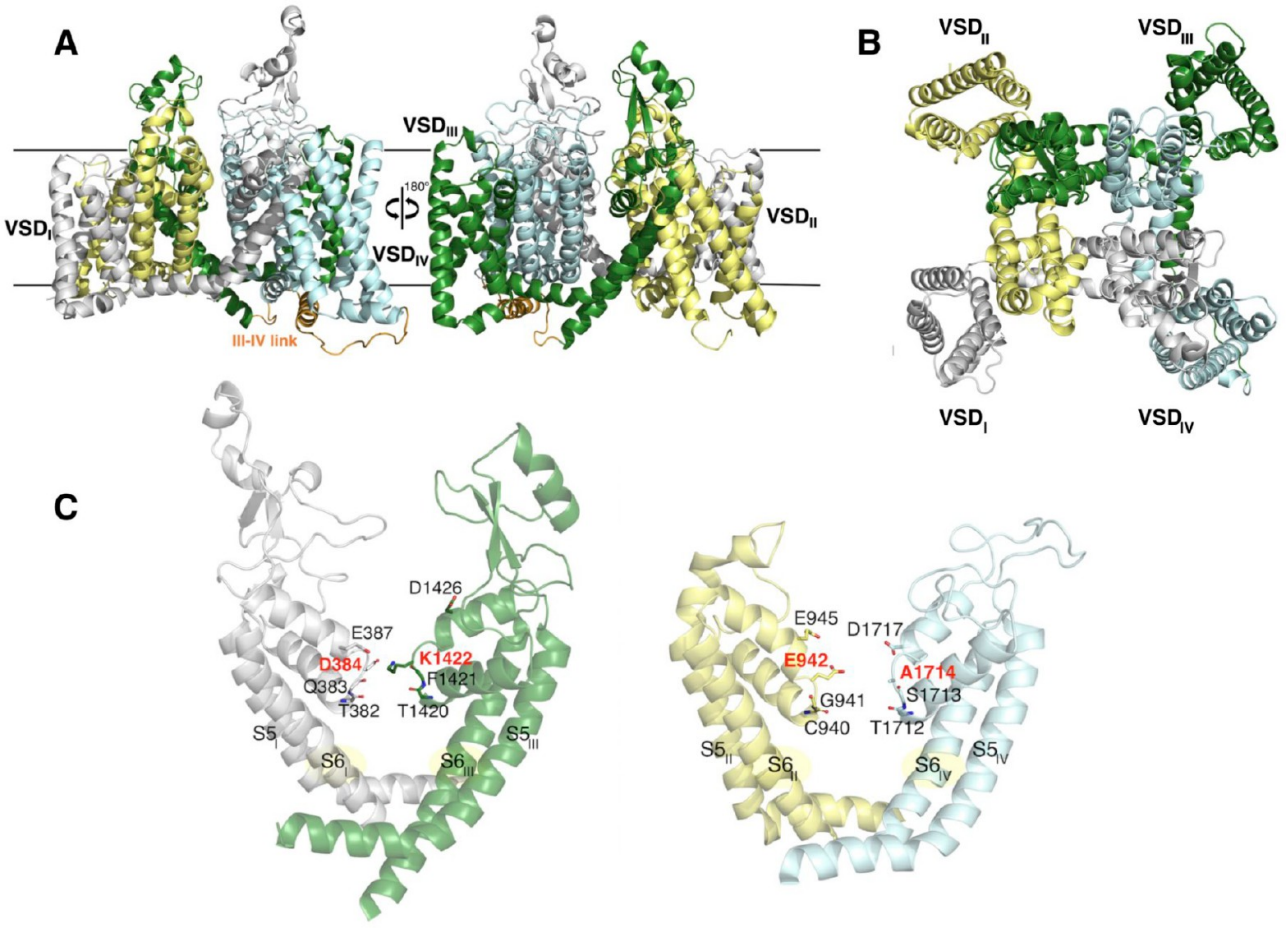
(A) Equilibrated homology-based Na_v_1.2 model. The domains are colored as in refs (63−65): Domain I, gray; Domain II, yellow; Domain III, green; Domain IV, cyan. This color scheme is applied throughout the article. (B) Extracellular view of the model. (C) Representation of the asymmetric Na_v_1.2 SF. The SF vestibule is enclosed by the side chains of the D_I_E_II_K_III_A_IV_ motif (D384, E942, K1422, A1714) and the internal carbonyl oxygen atoms of the two preceding residues in each repeat. The residues of the external E_I_E_II_D_III_D_IV_ motif (E387, E945, D1426, D1717), above the DEKA domain, are also shown. The EEDD domain and the SF region form the C/SF.

**Figure 2:**
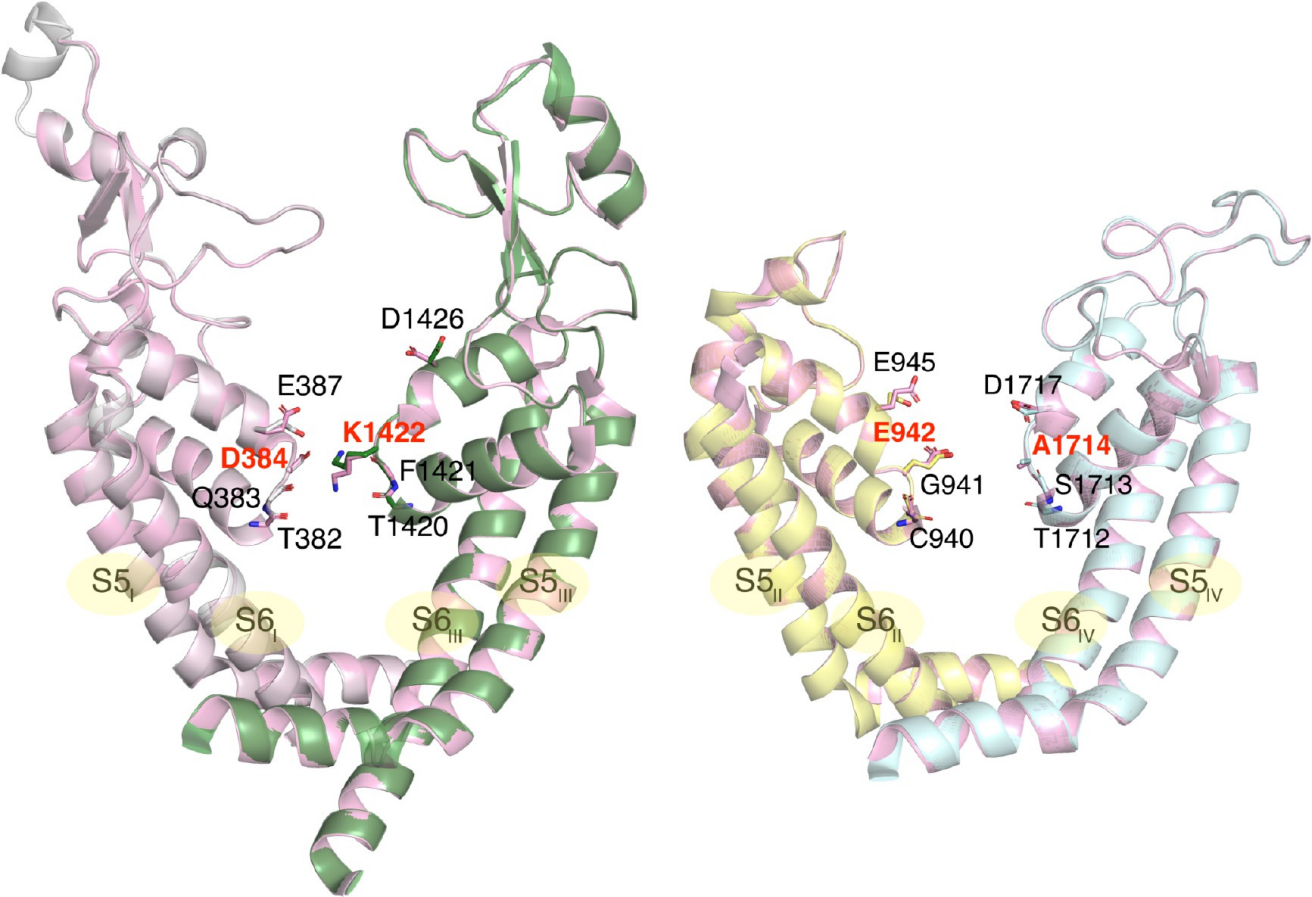
Superposition between the pore of the template (PDB ID 6AGF, hNa_v_1.4, pink ribbons) and the equilibrated homology-based Na_v_1.2 model. The domains are colored according to the convention introduced in Figure 1. The side chains belonging to the C/SF are shown as sticks. The residues are labeled following the Na_v_1.2 enumeration.

The α subunit of human Na_v_ channel is formed by four TM domains linked by cytoplasmic loops. Our TM structural models of human neuronal Na_v_ channels were constructed in two steps following a procedure similar to the one introduced in refs (106) and (107). First, we modeled each of the four TM domains separately using the advanced protein prediction method included in the SWISS-MODEL server,^108^ with the exemption of the Rep. DIII and Rep. DIV. These last domains were modeled together, including also the cytosolic III–IV link. The conservation between the sequence of the template and the domains in each of the three neural Na_v_ channels spans from ∼85% to ∼91%. Then, at a second stage, the whole subunit for each channel was built by structurally aligning the four domains to the template in a clockwise order viewed from extracellular side, using UCSF Chimera.^109^ The resultant tetrameric model was refined again by FG-MD^110^ to eliminate unwanted interdomain steric clashes and improve the global model quality. Before MD simulations, all the systems were aligned to the membrane normal axis *z* using the OPM-PPM^111^ service available at opm.phar.umich.edu/ppm_server. We show the homology-based Na_v_1.2 model in Figure 1, including the representation of the asymmetric Na_v_1.2 SF. We also show the superposition of the homology-based Na_v_1.2 model and the template (Figure 2) to confirm the conservation of the C/SF region. The Na_v_1.1 and Na_v_1.6 SFs are also shown in Figures S1 and S2, respectively.

#### MD of the WT Homology-Based Na_v_1.2 Model: Standard Atomic Masses Setup

We performed a 500 ns long MD simulation of the human Na_v_1.2 (hNa_v_1.2) homology-based model to assess the quality of the modeling protocol. CHARMM hydrogen atoms were added with CHARMM-GUI, and disulfide bonds were assigned according to the information provided in the PDB structures 5XSY, 6AGF, and 6J8E. In particular, the disulfide bond between C950 and C959, which is located inside the pore cavity, was included in all the simulations of this work. The SF filter was modeled with a DEK^+1^A ring. Then the Na_v_1.2 structure was inserted in a homogeneous lipid patch of 1-palmitoyl-2-oleoyl-*sn*-glycero-3-phosphocholine (POPC) molecules with explicit water molecules, and the total charge was neutralized with a 150 mM NaCl solution, obtaining ∼290,000 atoms in total. For this MD simulation, as for the other standard MD runs described in this work, we used the NAMD software^112^ (version 2.12) and the CHARMM36m^113−115^ and CHARMM36^116^ parameters for the protein and lipids, respectively, together with TIP3P model for water molecules^117^ and the associated ionic parameters with NBFIX corrections.^118−120^ Tetragonal periodic boundary conditions (PBCs) were applied to the simulation box to remove surface effects. Long-range electrostatic interactions were calculated using the particle mesh Ewald (PME) algorithm.^121^ Electrostatic and van der Waals interactions were calculated with a cutoff of 12 Å and the application of a smoothing decay starting to take effect at 10 Å.

In this specific MD run, a time step of Δ*t* = 2 fs was employed. To ensure maximum accuracy, electrostatic and van der Waals interactions were computed at each simulation step. All covalent bonds involving hydrogen atoms were constrained using the SHAKE and SETTLE algorithms.^122,123^ Before production, the system was relaxed following an equilibration for ∼35 ns by extending the CHARMM-GUI equilibration protocol in order to allow proper hydration of solvent-exposed regions of the Na_v_ pore cavity. Then the system was simulated in the *NPT* ensemble using the Nosé–Hoover Langevin piston method^124,125^ to maintain the pressure at 1 atm and a Langevin thermostat to maintain the temperature at 310 K. The oscillation period of the piston was set at 50 fs and the damping time scale at 25 fs. The Langevin thermostat was set with a damping coefficient of 1 ps^–1^.

#### MD of the WT Homology-Based Na_v_1.1, Na_v_1.2, and Na_v_1.6 models: HMR Setup

The three neuronal models Na_v_1.1, -1.2, and -1.6 were simulated in the WT configuration. In all these standard simulations, the K residue (K1422 in the Na_v_1.2 channel) was studied in the charged configuration (DEK^+1^A), and the hydrogen mass repartitioning (HMR) method was used to build the topology of the system. HMR is a computational methodology that redistributes the atomic masses.^126,127^ Thanks to this scaling, a larger time step can be used in the simulations. The method was originally proposed in ref (126) and then successfully applied to protein simulations in ref (127) using a hydrogen mass of 3 amu and a Δ*t* of 4 fs. For our simulations, CHARMM hydrogen atoms were added with CHARMM-GUI, and disulfide bonds were assigned as done for the previous system, including the disulfide bond between C950 and C959 in Na_v_1.2 (C959–C968 in Na_v_1.1 and C944–C953 in Na_v_1.6, respectively), which is located inside the pore cavity. Then the Na_v_ structures were inserted in a POPC membrane and solvated with explicit water molecules and a 150 mM NaCl solution. For all these MD simulations, we used the NAMD software^112^ and the parameters of the previous simulation with standard atomic masses. Tetragonal PBCs were applied to the simulation box to remove surface effects. Long-range electrostatic interactions were calculated using the PME algorithm.^121^ Electrostatic and van der Waals interactions were calculated with the standard CHARMM cutoff of 12 Å and the application of a smoothing decay starting to take effect at 10 Å following the instructions in ref (128).

In these HMR simulations, a time step of 4 fs was employed coupled to the HMR scheme. Electrostatic and van der Waals interactions were computed at each simulation step. All covalent bonds involving hydrogen atoms were constrained using the SHAKE and SETTLE algorithms.^122,123^ Before the production, the systems were relaxed following an equilibration for ∼35 ns. Then, they were simulated in the *NPT* ensemble using the Nosé–Hoover Langevin piston method^124,125^ to maintain the pressure at 1 atm and a Langevin thermostat to keep the temperature at 310 K. The oscillation period of the piston was set at 300 fs and the damping time scale at 150 fs.^128^ The Langevin thermostat was employed with a damping coefficient of 1 ps^–1^.

#### MD of the WT Cryo-EM Na_v_1.2 Structure: HMR Setup

**Figure 3:**
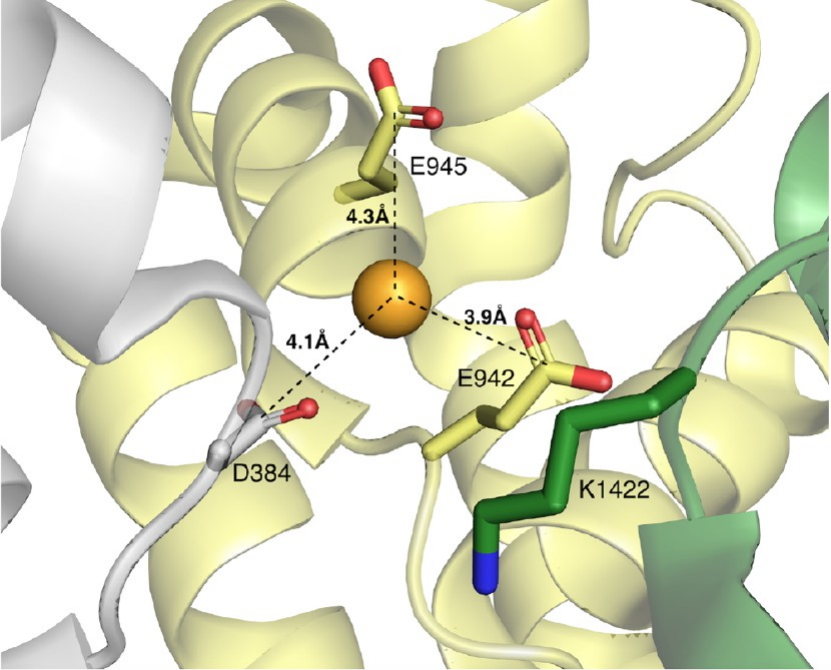
Sodium ion resolved in the SF of the PDB ID 6J8E Na_v_1.2 cryo-EM structure. The pore blocker μ-conotoxin KIIIA is not shown.

We used also an experimental cryo-EM structure of the human Na_v_1.2 channel. The channel is bound to the peptidic μ-conotoxin KIIIA pore blocker (PDB ID 6J8E).^99^ Notably, the structure includes a Na^+^ ion in the C/SF, as illustrated in Figure 3. Moreover, the bottom tip of the KIIIA peptide includes only one residue, K7, whose amine group interacts with E945.^99^ The overall conformation of the experimental-based Na_v_1.2 is substantially identical to the Na_v_1.4 template with an RMSD of 0.696 Å over 981 Cα atoms, according to the analysis exposed in ref (99). Based on this observation, this structure nicely fits with our homology-based Na_v_1.2 model. The superposition of the two structures is illustrated in Figure S3. The comparison between the cryo-EM Na_v_1.2 structure and our preliminary homology-based Na_v_1.2 model is characterized by an RMSD of 0.746 Å over the Cα atoms belonging to the TM α helices. Furthermore, the pore-only configuration of the new Na_v_1.2 structure differs from our pore-only model by an RMSD of 0.534 Å. To further investigate the features of this new structure, we produced two MD runs of the α subunit of this structure (after removing all the non-α-subunit atoms, including the toxin and the ion). In particular, we performed two 500 ns MD simulations with a physiological 150 mM NaCl concentration using the HMR protocol illustrated in the previous paragraph. The RCD+ loop closure tool^129,130^ was used to link the adjacent residues in the missing region of the long extracellular DI loop in order to form a single DI polypeptide chain, as previously done in the homology-based models.

#### Cross Distances Analysis and Ion Permeation Events

We calculated a set of distances between atoms of opposite repeats, reported in Tables S1−S4. For each standard MD run of the whole systems (VSDs + pore) listed in Table S5, we monitored the ionic permeation events through the SF in the presence of a physiological concentration of 150 mM of NaCl. The calculation includes the volume of the C/SF. Here we report the details of the selections using the notation of the Na_v_1.2 residues. Similar selections were made for the Na_v_1.1- and Na_v_1.6-based systems. The selected domain is characterized by the following features:

- a radius of 7 Å around the pore axis;
- the level of the upper circular section is defined by the *z* coordinate of the Cα atom following the E939 residue of the EEDD domain;
- the level of the lower circular section is defined by the *z* coordinate of the Cα atom of residue 940, further reduced by 4 Å.

#### Distributions of Atomic Positions

We analyzed the positions within the C/SF of one sodium ion and the lysine side chain of the DEKA motif. To this goal, we combined all six MD runs of the WT systems in NaCl solution, for a total of 3 μs. For each dataset, we reconstructed the histogram of values of the *z* coordinate of a single Na^+^, provided that they were found inside the C/SF. The same analysis was performed for the nitrogen atom belonging to the DEKA lysine’s side chain, which is supposed to play a role in ionic permeation through mammalian Na_v_ channels.^46,85−90^ The aggregation of the results from various systems is justified by the fact that the homology-based Na_v_1.2 model and the cryo-EM Na_v_1.2 structure are nearly identical in the C/SF and, because this is a highly conserved region in the various Na_v_ proteins, the identity is transferred also to the other channels we built. Therefore, in terms of this analysis, we considered the various neuronal Na_v_ channels as equivalent systems.

#### Additional MD of the WT Cryo-EM Na_v_1.2 Structure with Two Na^+^ Confined in the C/SF: HMR Setup

To further investigate the two-ion dynamics in the C/SF, we performed a set of additional standard MD simulations of the WT cryo-EM Na_v_1.2 structure (pore only) with two Na^+^ confined in the C/SF by applying a set of half-harmonic restraints. The cryo-EM pore was prepared using the membrane builder plugin of the CHARMM-GUI web server, embedded in a POPC bilayer and solvated with the TIP3P water model and a physiological NaCl concentration of 150 mM. Aiming at the hydration of the pore cavities, a preliminary equilibration stage of 20 ns was accomplished using the input files generated by CHARMM-GUI with the same setup as the previous simulations. In the equilibrated structure at *t* = 20 ns, two Na^+^ ions were dragged into the C/SF from the solvent in order to obtain three starting configurations accounting for two ions randomly positioned in the C/SF domain. Each configuration of the system was further subjected to an additional equilibration phase of ∼40 ns. During this stage, positional restraints were introduced on the protein with a gradually softened strength, so that in the last 10 ns only the α carbons of the S5 and S6 segments were unable to move, while P-loops were maintained free throughout the sampling. The two Na^+^ ions located in the C/SF region were confined by the application of two κ = 100 kcal/mol half-harmonic restraints at *z* > 25 Å and *z* < −2 Å. Simultaneously, the access to the C/SF was prevented to other ions by the application of a repulsive restraint which applies in a volume positioned approximately at the center of the DEK^+1^A motif by adding the following biases to the MD potential:
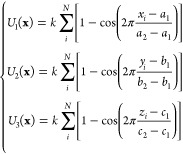
1where **x** = {*x*, *y*, *z*} are the coordinates of all the atoms in the system and the summations run over the cations to be excluded from the SF, i.e., from the volume delimited by {*x*_min_, *y*_min_, *z*_min_} = {−10 Å, −10 Å, −20 Å}, and {*x*_max_, *y*_max_, *z*_max_} = {10 Å, 10 Å, 25 Å}. In eqs 1, *a*_1_ = −10 Å, *a*_2_ = 10 Å, *b*_1_ = −10 Å, *b*_2_ = 10 Å, *c*_1_ = −20 Å, *c*_2_ = 25 Å, and *k* = 600 kcal/mol. This potential introduces a prohibitive barrier for the excluded molecules to enter the SF. For each of the equilibrated configurations, four replicas were simulated (REPLICA1–4), for a total of 3 × 4 = 12 MD runs. In REPLICA4, the exclusion volume is not applied, while the confinement of the two ions along the *z* axis is preserved. Every system was simulated for 100 ns in the *NPT* ensemble with the same parameters used during the 40 ns-long equilibration stage. The cumulative sampling amounts to 1.2 μs.

### TAMD/OTFP Simulations for Free Energy Calculations

The free energy (FE) of an *N*-atom system, with potential energy *U*(**x**) and at temperature *T*, is defined as
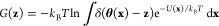
2where the integral runs over the system configurations **x** ∈  and **z** ∈  represents a point in the lower-dimensional space of collective variables (CVs) obtained via the transformation **θ**:  → , with *M* < 3*N*. In temperature-accelerated molecular dynamics (TAMD),^95^ a set of *M* auxiliary variables **z** = *z*_1_, ···, *z*_*M*_ are introduced and tethered to the *M* CVs **θ** = θ_1_, ···, θ_*M*_. The combined set (**x**, **z**) is subjected to the following potential:
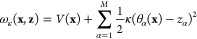
3where *V*(**x**) is the MD force field, and κ > 0 is an adjustable spring-constant-like parameter. The extended system evolves according to coupled equations, as for example:
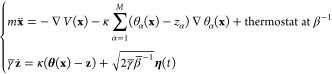
4where *m* is the mass, **η**(*t*) is a Gaussian process with mean 0 and covariance ⟨η_α_(*t*)η_α′_(*t*′)⟩ = δ_αα′_δ(*t* – *t*′), γ̅ is a friction coefficient, and 1/β̅ is an artificial temperature with 1/β̅ > 1/β, where β = 1/*k*_B_*T*. As shown in ref (95) (see also ref (131)), by adjusting the parameter κ so that **z** ∼ **θ**(**x**(*t*)) and the friction coefficient γ̅ so that **z** moves slower than **x**, a trajectory **z**(*t*) can be obtained which moves at the artificial temperature 1/β̅ on the FES calculated at the physical 1/β. Indeed, it has been demonstrated^95^ that under such assumptions each auxiliary variable *z*_*j*_ is driven by the force
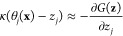
5where *G*(**z**) is given by eq 2. Hence, by choosing 1/β̅ > 1/β in eq 4, the **z** trajectory will rapidly visit the regions where the FE is relatively low, overcoming barriers that the system would take a long time to cross at the physical temperature 1/β. In addition, the on-the-fly free energy parametrization (OTFP) method is an efficient approach to reconstruct the FES sampled by a TAMD simulation.^96−98^ The idea is to introduce an approximation of the FE via a set of basis functions ϕ_*m*_(**z**):
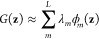
6and to determine the coefficients in eq 6 by minimizing the following error function:^132^
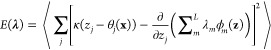
7where λ is the  vector of the λ_*m*_ coefficients and ⟨ ⟩ indicates the average computed during the TAMD simulation. This is equivalent to solving a linear system of equations **Aλ** = **b**, where
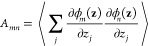

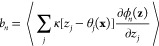
8Following refs (96−98) and (100), chapeau functions are used as basis set. Other FE reconstruction methods based on the minimization of the same error function have been introduced more recently.^133,134^

#### TAMD/OTFP Simulations: Standard Atomic Masses Setup

**Figure 4:**
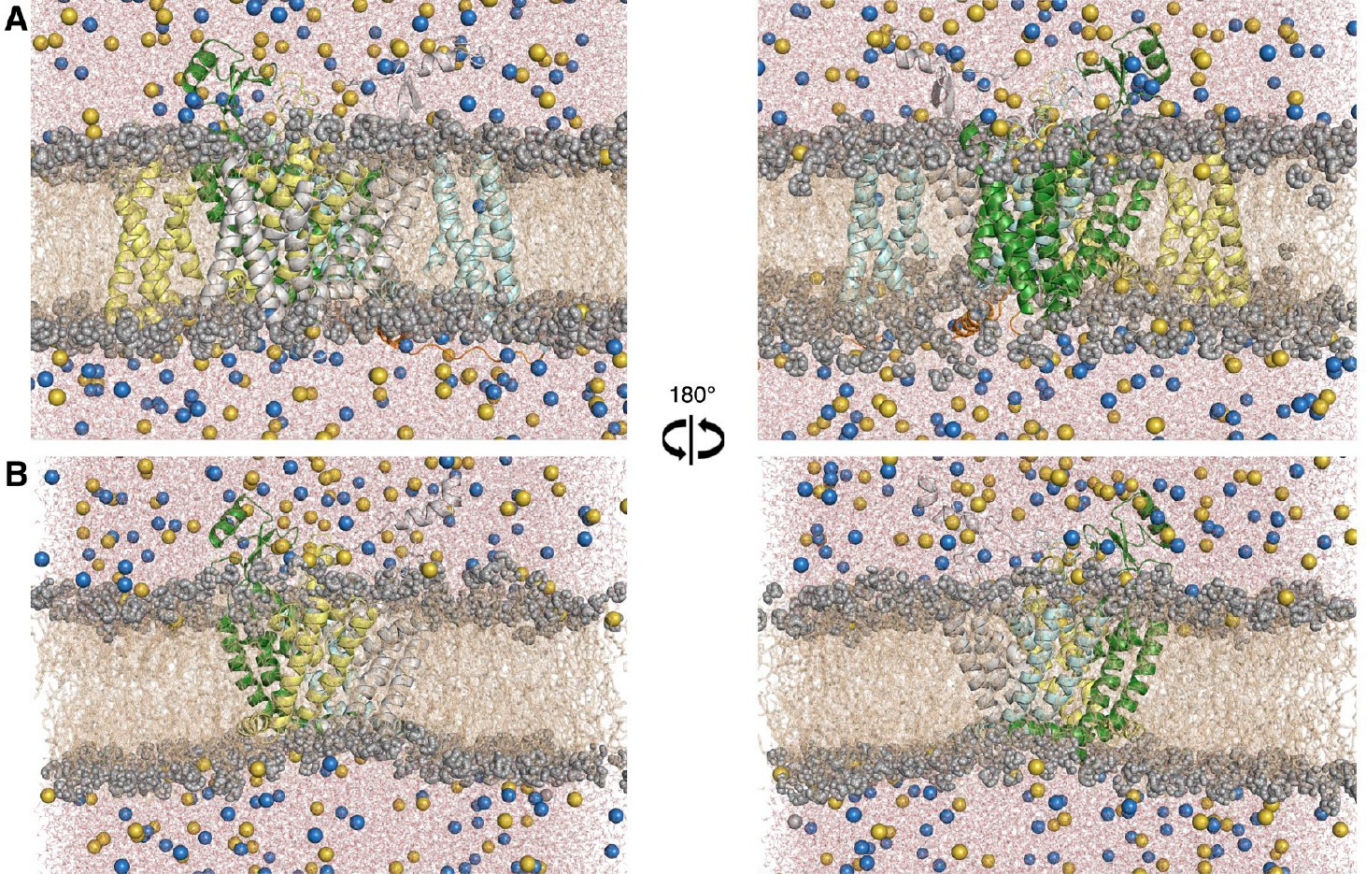
(A) Equilibrated homology-based Na_v_1.2 α subunit embedded in a hydrated POPC bilayer (brown wires), surrounded by water (red and white). Sodium (yellow) and chloride (blue) ions are represented as spheres. (B) Equilibrated homology-based Na_v_1.2 pore-only domain embedded in the same environment as described in (A).

The NAMD code^112,135^ was used to integrate the atomic equations of motion in the OTFP simulations, while the auxiliary equations and the reconstruction were handled by a C code via the NAMD TcL forces plugin,^98,136^ available at github.com/cameronabrams/otfp. The reduced pore-only configuration of the former Na_v_1.2 model used for the TAMD/OTFP calculations includes the following residues: Rep. DI, from L234 to A427, with the exclusion of residues from N285 to T305; Rep. DII, from T866 to L983; Rep. DIII, from F1320 to F1476; Rep. DIV, from K1641 to I1775. The protein was aligned to the membrane normal *z* axis using OPM-PPM.^111^ The same pore configuration was obtained from the cryo-EM Na_v_1.2 structure (PDB ID 6J8E). In order to use absolute coordinates as CVs for both systems, they were aligned by superimposing the cryo-EM pore on the model one. As reference, the *z* coordinate of the cryo-EM K1422 NZ atom after alignment is 6.8 Å. The resolved Na^+^ ion in the SF (at *z* = 12.7 Å) was removed from the PDB file. Then, both systems were inserted in a homogeneous POPC membrane and solvated with explicit water molecules, and the total charge was neutralized with a 150 mM NaCl solution. The minimum starting size of the periodic box measured ∼ [135 × 135 × 129 ] Å^3^, which ensured a distance larger than 20 Å between adjacent images of the protein during the simulations. For all the TAMD/OTFP simulations, we employed the CHARMM parameters already used for the standard MD simulation with original masses. Tetragonal PBCs were applied to the simulation box, and long-range electrostatic interactions were calculated using the PME algorithm.^121^ Electrostatic and van der Waals interactions were calculated with a cutoff of 12 Å and the application of a smoothing decay starting to take effect at 10 Å. A time step of 2 fs combined with the SHAKE and SETTLE algorithms^122,123^ was used. In order to ensure maximum accuracy, electrostatic and van der Waals interactions were computed at each simulation step. Before production, the system was equilibrated for ∼25 ns. A snapshot of the equilibrated system is illustrated in Figure 4B. During all the TAMD/OTFP runs, soft harmonic restraints were maintained on the Cα atoms of each α S5–S6 helix in order to avoid any rigid-body rotational or translational displacement of the protein. More specifically, C_α_ atoms of the residues from L234 to M271 and from K399 to A427 in Rep. DI, residues from T866 to Y907 and from Q956 to L983 in Rep. DII, residues from E1321 to F1359 and from L1447 to F1476 in Rep. DIII, and residues from A1643 to F1682 and from P1749 to I1775 in Rep. DIV were restrained. On the other hand, no restraints were applied to the C/SF atoms. After the preparation, the systems were simulated in the *NPT* ensemble using the Nosé–Hoover Langevin piston method^124,125^ to maintain the pressure at 1 atm, and a Langevin thermostat to maintain the temperature at 310 K. The oscillation period of the piston was set at 50 fs and the damping time scale at 25 fs. The Langevin thermostat was employed with a damping coefficient of 1 ps^–1^. The ionization state of the protein residues corresponds to the neutral pH (DEK^+1^A and DEE^–1^A), except for the simulations that include the modified protonation of the K/E1422 residue (DEK^0^A and DEE^0^A). All the runs where performed in a zero-transmembrane-voltage regime. All the cations not involved in the definition of the CVs were excluded from the C/SF by applying a repulsive restraint. This restraint applies in a rectangle positioned approximately at the center of the DEK(E)A motif by including the following bias to the force field expression of the potential energy:
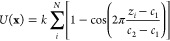
9where the summation runs over the excluded cations inside the SF, i.e., inside the rectangle delimited by the coordinates {*x*_min_, *y*_min_, *z*_min_} = {−6 Å, −6 Å, −4 Å}, and {*x*_max_, *y*_max_, *z*_max_} = {6 Å, 6 Å, 18 Å}, which was used for the majority of the simulations, as reported in Table S6. Here, *c*_1_ = −4 Å, *c*_2_ = 18 Å, and *k* = 600 kcal/mol. This potential introduces a prohibitive barrier for the excluded molecules to enter the SF. Since this volume is contained within the transmembrane domain of the channel, biases on *x* and *y* are not needed to avoid the lateral permeation of additional Na^+^ ions. The same potential term was previously used in ref (98) to perform TAMD/OTFP simulations of a representative voltage-gated potassium (K_v_) channel while keeping water molecules outside the K_v_ SF. The single or two ions used for defining the CV were confined inside the filter by the application of two 300 kcal/mol half-harmonic potentials at *z* > 17 Å and *z* < 4 Å. Two-dimensional (2D) FESs were recovered by combining the following CVs:

- *z*_1_: the *z* coordinate of one Na^+^ in the C/SF. This CV will be denoted as *r*_*z*_(Na_1_).
- *z*_12_: the *z* coordinate of the center of mass (COM) of two Na^+^ ions in the C/SF. This CV will be denoted as COM_*z*_(Na_1_, Na_2_).
- *z*_NZ_: the *z* coordinate of the nitrogen atom (NZ in CHARMM notation) of the K1422 side chain. This CV will be denoted as *r*_*z*_(K-NH_3_^+^) in the DEK^+1^A and *r*_*z*_(K-NH_2_) in the DEK^0^A, respectively.
- *z*_CD_: the *z* coordinate of the carbon δ atom (CD in CHARMM notation) of the E1422 side chain. This CV will be denoted as *r*_*z*_(E-COO^–^) in the DEE^–1^A and *r*_*z*_(E-COOH) in the DEE^0^A, respectively.

In the following, we will describe two sets of TAMD/OTFP simulations. The first, denoted with OTFP_1_, considers *z*_1_ and either *z*_NZ_ or *z*_CD_ depending on the composition of the 1422 residue. The second one, denoted with OTFP_2_, considers *z*_12_ and either *z*_NZ_ or *z*_CD_. The auxiliary temperature was 6000 K, the auxiliary friction 800 (kcal/mol) ps/Å^2^, and the spring constant 1500 kcal/mol Å^–2^, following ref (98). The OTFP_1_ and OTFP_2_ setups were produced for all the SF configurations included in this work (DEK^+1^A, DEK^0^A, DEE^–1^A, and DEE^0^A) in the presence of a 150 mM NaCl concentration using both the homology-based Na_v_1.2 model and the cryo-EM Na_v_1.2 structure. A list of the OTFP simulations is reported in Table S6.

#### String Method

Using the string method,^137−139^ we computed the minimum free energy path (MFEP) over the averaged DEK^+1^A surface. The initial string was generated using the union of two linear interpolations, one between (5.0, 11.4) and (3, 10) (8 points) and the other between (3.0, 10.0) and (15.0, 7.8) (50 points). The string method was implemented with a MATLAB script, using finite differences to obtain forces from the FES. A tolerance of 10^–7^ was used as convergence threshold for the mean deviation between two consecutive string parametrizations. The FE profile along the MFEP was obtained by using the free energy values of the MFEP points in the FES.

#### TAMD/OTFP Simulations: HMR Setup

In order to further test the convergence of our FESs, we also performed longer simulations using the cryo-EM Na_v_1.2 (OTFP_1_ DEK^+1^A) with the HMR setup. Three runs (REPLICA1/2/3) based on the same protocol of the standard HMR MD simulations, using an *NPT* ensemble maintained by the Nosé–Hoover Langevin piston method^124,125^ to keep the pressure at 1 atm and a Langevin thermostat to maintain the temperature at 310 K. Here, the oscillation period of the piston was set at 300 fs and the damping time scale at 150 fs.^128^ The Langevin thermostat was employed with a damping coefficient of 1 ps^–1^. Two additional runs were performed (REPLICA4/5) using a Langevin damping coefficient of 0.5 ps^–1^ (in REPLICA4 and REPLICA5) and a number of steps between updates of the nonbonded pair list of 5 (in REPLICA5) instead of 20, as suggested by ref (128) for improved accuracy.

#### Structural Analysis and Atomic Density Calculations

All the MD trajectories were visualized and analyzed using UCSF Chimera^109^ (www.cgl.ucsf.edu/chimera/), PyMOL^140^ (pymol.org/2/), NAMD-COLVAR,^141^ and VMD^142^ (www.ks.uiuc.edu/Research/vmd/) with in-house Tcl scripts. The VMD QuickSurf representation^143−145^ was used to represent the volume occupancy of selected atoms in the metastable conformations extracted from the TAMD/OTFP simulations. This VMD plugin computes an isosurface extracted from the expansion of a Gaussian density kernel map at each particle position and uses a 3D grid to sum all together and get the final density. Volume densities were colored according to charged atoms from DEKA and EEDD residues (red for all the negatively charged residues). The density of the K/E1422 residue is represented according to the specific system (blue for DEK^+1^A, cyan for DEK^0^A, red for DEE^–1^A, orange for DEE^0^A). Na^+^ density is also included (blue). Snapshots of the equilibrated full and pore-only Na_v_1.2 model conformations are shown in Figure 4.

## Results and Discussion

**Figure 5:**
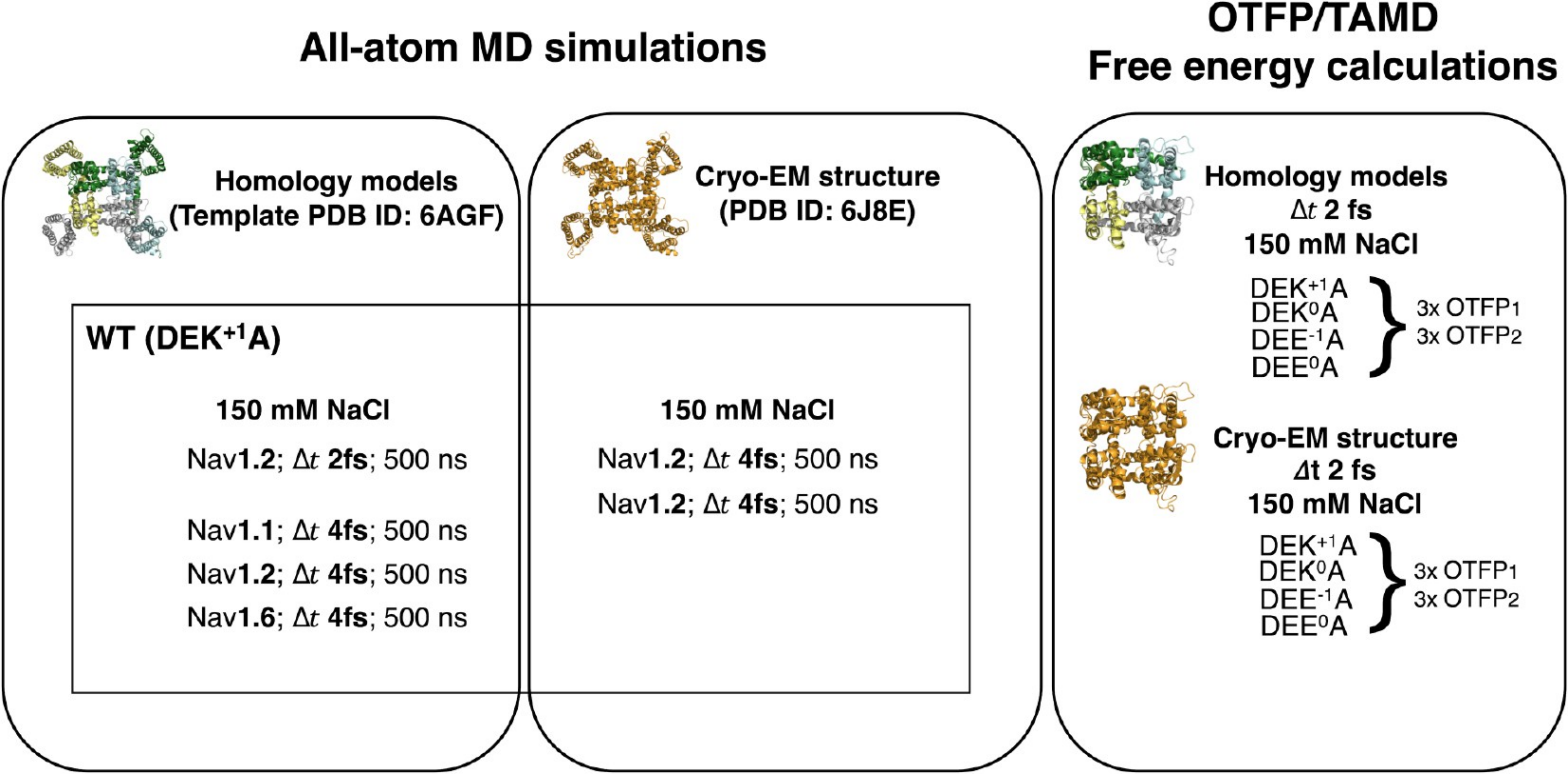
Main simulations performed in this work. For each MD run, the integration time step Δ*t* is shown. The meaning of OTFP_1_ and OTFP_2_ is specified in the text. Results of additional simulations are included in the Supporting Information. The list of all the simulations included in this work is reported in Tables S5 and S6.

This section is organized in two parts. In the first one, we report the results obtained from the standard MD simulations, in terms of both assessment of the quality of the different structures and the evaluation of the observed Na^+^ permeation events through the SF. In the second subsection, we describe the thermodynamic features of Na^+^ translocation into the Na_v_1.2 SF by analyzing the 2D FESs extracted from the TAMD/OTFP simulations outlined in Materials and Methods. The list of all the simulations included in this work is reported in Figure 5 and in Tables S5 and S6.

### Standard MD Simulations of the Na_v_ Systems

#### Structural Stability of the Homology-Based Na_v_ Models and the Cryo-EM Na_v_1.2 Structure

We tested the stability of all the Na_v_ structures involved in this study via unbiased MD simulations. For each system, we calculated the backbone RMSD of the transmembrane pore domain, including the S5 and S6 helices of each repeat (Figure S4). All the RMSD profiles show plateaus at values of ∼2 or 3 Å, thus preserving the overall pore conformation. Moreover, we calculated the RMSD of the P-loops of the different structures in each simulation in order to check the stability of the SF. These additional calculations (shown in Figure S5) confirm the stability of the C/SF domain. Not surprisingly, we found no significant difference between the homology-based model and the cryo-EM structure of the Na_v_1.2 channel. We also calculated a set of cross distances (CDISTs), each defined between two conserved atoms or groups of atoms belonging to opposite repeats and listed in Tables S1–S4. The average values of all CDISTs, obtained from their mean in each MD replica run, are reported in Table S7. Results for the homology-based models and the cryo-EM structure are similar. Here we introduce only the CDISTs belonging to the EEDD (d1 and d2) and the DEKA (d3 and d4) motifs, defined as follows and whose values are reported in Table 2:

- **d1A**: E387CA–D1426CA; **d1B**: E387CD–D1426CG
- **d2A**: E945CA–D1717CA; **d2B**: E945CD–D1717CG
- **d3A**: D384CA–K1422CA; **d3B**: D384CG–K1422CE
- **d4A**: E942CA–A1714CA; **d4B**: E942CD–A1714CB

Here atoms are defined in the CHARMM notation:

- CA is the C_α_ atom of the backbone for each residue;
- CD, CG, and CE are the C_δ_, C_γ_, and C_ε_ atoms of glutamate, aspartate and lysine, respectively, i.e., the most external carbon atoms in the side chains of these residues.

**Table 2:** Summary of the Cross Distances in the C/SF Domain, Measured in the Cryo-EM Na_v_1.2 Structure (PDB ID 6J8E; Second Column) and as the Average among the Mean Values of the Single Standard MD Simulations (Third Column)

Cross Distance	PDB ID 6J8E (Å)	μ ± σ (Å)
d1A	17.90	17.06 ± 0.37
d1B	13.20	12.50 ± 0.40
d2A	17.60	19.37 ± 1.04
d2B	12.00	13.88 ± 1.01
d3A	10.80	10.45 ± 0.64
d3B	9.10	8.60 ± 0.57
d4A	11.90	12.13 ± 0.90
d4B	10.20	10.94 ± 0.81

The four CDISTs show an excellent conservation along the MD runs and confirm the stability of the C/SF filter and of the canonical initial position of the P-loops, as revealed also by the RMSD values shown in Figure S5. Selections of the residues for these additional RMSD calculations are listed in Table 3.

**Table 3:** List of All the Residues Forming the P-Loop Domains and Included in the RMSD Calculations for the Backbone of the SF; The Starting UNIPROT IDs (UIDs) with the Associated Human Sequences Are Included

Channel	Na_v_1.1 (UID: P35498)	Na_v_1.2 (UID: Q99250)	Na_v_1.6 (UID: Q9UQD0)
Rep. DI	F368–A395	F370–A397	D354–A383
Rep. DII	F938–V962	F929–G955	F922–V947
Rep. DIII	N1417–D1443	V1408–S1434	V1399–D1424
Rep. DIV	F1710–L1734	F1700–L1724	T1690–I1714

#### Interactions between Sodium Ions and the C/SF

Despite the limited sampling of our unbiased MD simulations, they can still provide a partial picture of Na^+^ translocation through the filter. We thus analyzed ion occupancy in the C/SF region, as defined in Materials and Methods. In all the standard MD simulations, we observed various ion permeation events that produced the ionic occupancies reported in Table 4. While all Na_v_ systems prefer single Na^+^ occupancy, they also show a small percentage of two-Na^+^ configurations, as further confirmed by the results exposed in Table 5, where we report the average Na^+^ occupancy during each standard MD run.

**Table 4:** Summary of the Ionic Occupancies (from 0 to 4) in the C/SF during the Standard MD Simulations^a^

Na_v_ (SF)	System	Ions	Time (ns)	0 (%)	1 (%)	2 (%)	3 (%)	4 (%)
1.2 (DE**K**^+1^A)*	Model	[NaCl]	500	7.7	66.3	25.1	0.9	–
1.1 (DE**K**^+1^A)	Model	[NaCl]	500	15.6	65.1	19.0	0.3	–
1.2 (DE**K**^+1^A)	Model	[NaCl]	500	10.9	59.7	28.3	1.1	–
1.6 (DE**K**^+1^A)	Model	[NaCl]	500	7.6	65.2	26.7	0.5	–
1.2 (DE**K**^+1^A)	Cryo-EM	[NaCl]	500	4.3	53.4	41.1	1.2	–
1.2 (DE**K**^+1^A)	Cryo-EM	[NaCl]	500	18.5	73.1	8.0	0.4	–

a The quantities are expressed as percentages over the total amount of frames. All simulations were performed using the HMR protocol and a time step Δ*t* = 4 fs, except for the starred one, in which standard atomic masses and a time step Δ*t* = 2 fs were used.

**Table 5:** Average Na^+^ Occupancy in the C/SF of the Channels during Standard MD Simulations^a^

Na_v_ (SF)	System	Ions	Δ*t* (fs)	Time (ns)	Occupancy
1.2 (DE**K**^+1^A)*	Model	[NaCl]	2	500	1.19 ± 0.57
1.1 (DE**K**^+1^A)	Model	[NaCl]	4	500	1.04 ± 0.60
1.2 (DE**K**^+1^A)	Model	[NaCl]	4	500	1.19 ± 0.63
1.6 (DE**K**^+1^A)	Model	[NaCl]	4	500	1.20 ± 0.57
1.2 (DE**K**^+1^A)	Cryo-EM	[NaCl]	4	500	1.39 ± 0.59
1.2 (DE**K**^+1^A)	Cryo-EM	[NaCl]	4	500	0.90 ± 0.51

a The occupancy is introduced as average ± standard deviation. All simulations used the HMR protocol and Δ*t* = 4 fs except for the starred one, which employed Δ*t* = 2 fs and standard masses.

We then looked at the specific positions of the Na^+^ ion in the C/SF in all the frames that showed a single cation permeating the filter. These snapshots form the majority of the ionic configurations revealed by our MD runs. To this aim, we combined the single-ion configurations from all six MD runs of the different systems. The aggregation of results from the three Na_v_s is justified by the following observations:

1. The residues forming the C/SF domain are conserved across the three isoforms.
2. The starting, equilibrated structures of Na_v_1.1 and Na_v_1.6 have an RMSD of the P-loops less than or about 0.1 Å with respect to the analogous Na_v_1.2 conformation.
3. The RMSD values of the P-loops of all isoforms are stable during all the standard MD simulations (as shown in Figure S5), with maximum average value of 1.95 Å and maximum variance of 0.49 Å.
4. All isoforms show the same trend in terms of ion occupation in the C/SF domain, as shown in Tables 4 and 5.

**Figure 6:**
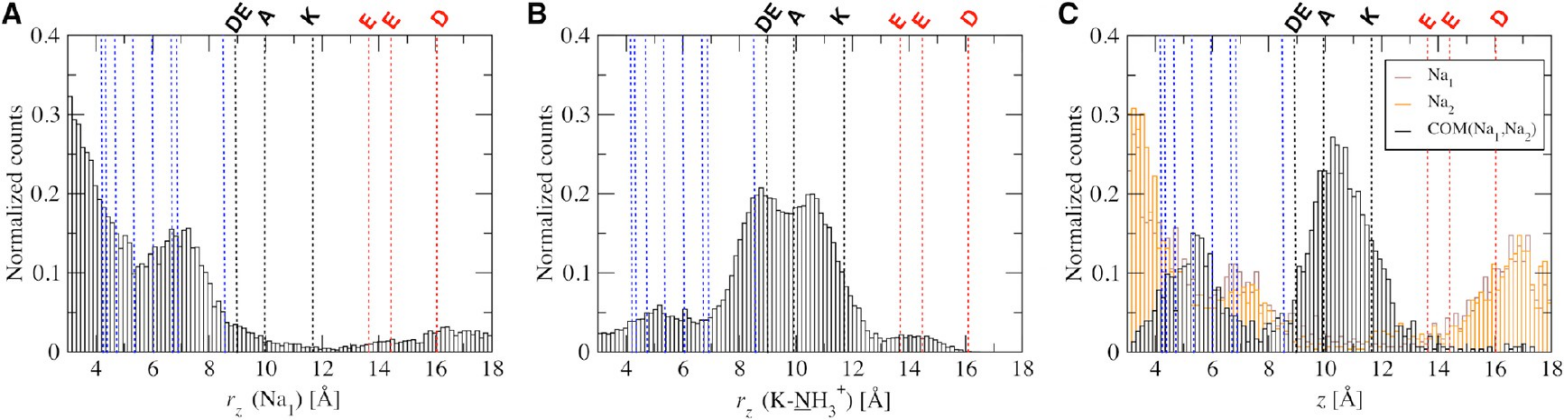
Histograms of the *z* coordinate values of (A) a single Na^+^ ion in the C/SF, (B) the ammonium nitrogen of the DEKA ring, and (C) two Na^+^ ions and their COM in the double occupancy events. Vertical lines indicate the *z* coordinates of the Cα atoms of C/SF residues from the equilibrated configuration of Na_v_1.2 (red for EEDD, black for DEKA, and blue for each couple of residues right below the DEKA ring).

In Figure 6 we show the histograms of the *z* coordinate of (A) the single Na^+^ ion in the C/SF and (B) the nitrogen of K1422 side chain, which is considered to play a relevant role in ionic permeation through mammalian Na_v_s.^46,85−90^ In addition, we show the histograms of the *z* coordinate of the two Na^+^ ions and of their center of mass (COM) in the cases of double occupancy. The interval of the *z* coordinate is between 3 and 18 Å, accordingly with the sampling of our TAMD/OTFP simulations (see below). In Figure S6A–C we also report the same histograms using an extended range of *z* coordinates, between −2 and 22 Å.

Results show that the single Na^+^ mostly occupies the lower portion of the SF, below the DEKA ring and in proximity of the inner rings of carbonyls. Conversely, the lysine side chain resides at a higher position along the axis, where the positive charge is expected to interact with the negatively charged residues of the ring. These interactions involving the lysine might even occlude the SF, competing with the passage of other alkali ions from the extracellular environment. A set of six movies showing different events of Na^+^ permeation throughout the C/SF and its residency in proximity of the lower rings are provided at https://github.com/BeatriceCorradi/Nav_Channels_movies.git. Our findings of single Na^+^ in proximity of the inner region of the SF are consistent with what was observed in ref (93) via MD simulations of multiple Na_v_ channels (including 1.2, 1.4, and 1.7) starting from cryo-EM structures. Notably, however, this position is different from the one occupied by a single Na^+^ ion in the Na_v_1.2 structure (PDB ID 6J8E). However, the cryo-EM structure captures the channel with the C/SF occluded by the bound μ-conotoxin KIIIA, which limits a comparison with our computational investigations.

Calculations for the *z* coordinates of the two Na^+^ ions in the case of double occupancy show the main peak of the COM at the center of the SF domain at ∼11 Å with one Na^+^ in proximity of the EEDD domain and the other in the internal part of the C/SF region below the DEKA motif. Because of the limited sampling of the two Na^+^ configurations in our standard MDs of whole channels, we performed a set of additional simulations of the pore-only configuration of the more recent cryo-EM Na_v_1.2 structure by confining two cations in the C/SF and calculated their positions along the *z* axis during a cumulative time of 1.2 μs. The resulting positional distributions confirm the peak for the COM occupancy at ∼11 Å and the associated positions of the ions, as shown in Figure S6D. The corresponding trajectories of the two ions and the lysine in the 12 simulations are reported in Figures S7–S9.

### 2D FES Calculations for Sodium Translocation through the Na_v_1.2 Selectivity Filter

We then used TAMD/OTFP simulations to reconstruct the thermodynamics of Na^+^ permeation through the C/SF of the Na_v_1.2 channel structures with different residue configurations, namely DEK^+1^A, DEK^0^A, DEE^–1^A, and DEE^0^A (see Materials and Methods). We performed two sets of TAMD/OTFP simulations, named OTFP_1_ and OTFP_2_, that differ in the definition of the CVs and describe, respectively, single- and two-Na^+^ translocation, the most probable spontaneous events observed in the standard MD simulations.

To investigate possible distortions in the pore-only configuration induced by the removal of the four VSDs, we also performed a standard MD simulation, with no restraints applied, of the cryo-EM pore-only structure (∼190 ns, HMR setup). The RMSD calculations shown in Figure S10 demonstrate that the deviation of the structure from its starting state is comparable to that of the whole channels (compare with Figure S4), even for the P-loops only (compare with Figure S5). These findings support the use of the pore-only configuration in our additional MD simulations with two ions confined in the C/SF (see previous section) and also in the accelerated simulations.

During all the OTFP/TAMD simulations, we monitored the stability of the C/SF domain by calculating the RMSD of the backbone for the residues belonging to the P-loops (according to the selection reported in Table 3). The calculations of the DEKA systems are shown in Figures S11 and S12 for the OTFP_1_ and the OTFP_2_, respectively. The calculations of the DEEA systems are illustrated in Figures S13 and S14, again for the OTFP_1_ and the OTFP_2_, respectively. They show a maximum average RMSD of 1.32 Å and maximum variance of 0.22 Å, in line with the data obtained from the standard MD simulations, demonstrating that the setup of the FE calculation (bias, effective temperature, exclusion volumes for other ions, absence of the VSDs) does not induce any distortion in the structure of the system.

In the next two paragraphs we show the average FESs obtained from multiple independent OTFP_1_ and OTFP_2_ runs, respectively, while results for the individual trajectories are reported in Figures S15–S18. All TAMD/OTFP runs are summarized in Table S6. In all simulations, the ions not involved in the CVs definition were excluded from the C/SF (see Materials and Methods). In order to investigate the effect of this setting, we performed a set of independent simulations for the DEK^+1^A system using a larger exclusion domain. Results are reported in Figure S19 and do not show any remarkable difference with the smaller domain.

Convergence was checked by comparing FESs obtained at different time lengths during the simulations. For instance, in Figure S20 we present the evolution of the FESs during a single replica simulation for the DEK^+1^A system. It is possible to see that the FES at 154 ns is very similar to the FES at 115 ns, and continuing the simulation did not result in major modifications. It is important to note that while this last figure corresponds to a single replica, the statistics of three simulations for the cryo-EM structure and three replicas for the homology model were combined to get the final FESs presented in the article, all based on the use of the standard atomic masses and the time step Δ*t* = 2 fs, in order to have maximum accuracy.

**Figure 7:**
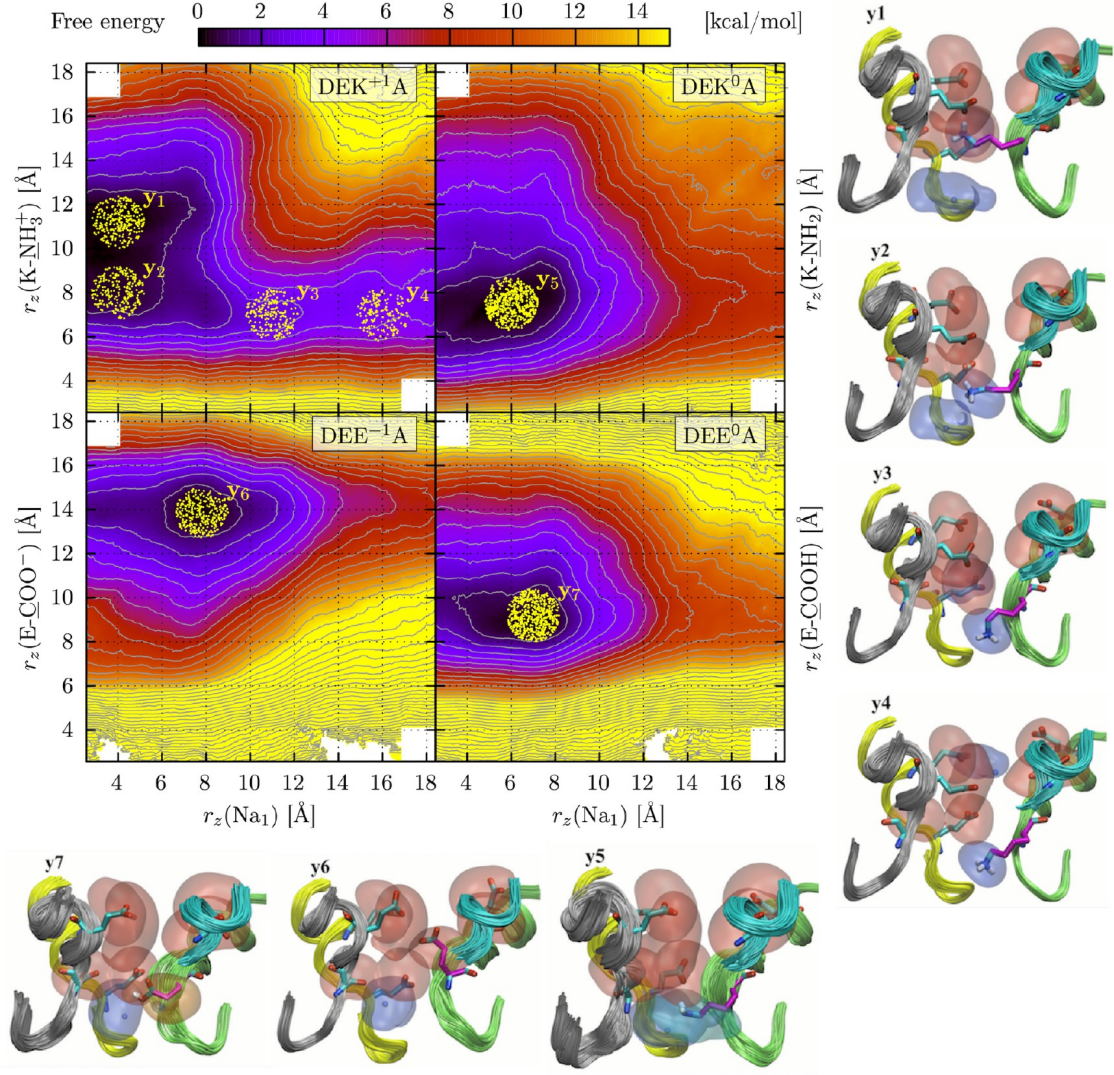
FESs for single Na^+^ translocation for each of the four systems, as indicated in the labels (DEK^+1^A, DEK^0^A, DEE^–1^A, DEE^0^A). Yellow dots indicate the sets of snapshots used for the representative configurations y_1_ to y_7_. For each set y_*i*_, the backbone of the various repeats is shown in ribbons, using the color code illustrated in Figure 1. For clarity, a fragment of Domain IV (cyan) is not drawn. The transparent surfaces represent the volume occupancy of Na^+^ and the tip atoms of the DEKA and EEDD side chains, and they are colored according to atomic charge (red for negative charges and blue for positive charges). The surface of residue 1422 is colored cyan for DEK^0^A and orange for DEE^0^A).

We also performed five HMR OTFP_1_ simulations of the cryo-EM Na_v_1.2 structure (DEK^+1^A SF), each for a time length of ∼300 ns. The associated FESs, shown in Figure S21, are very similar to those with standard atomic masses and Δ*t* = 2 fs (OTFP_1_ runs for the DEK^+1^A motif in Figure 7). Moreover, these simulations preserved the starting configurations of the P-loops, as confirmed by the RMSD calculations shown in Figure S22. Comparison of the total energy in the three replicas with standard atomic masses (Δ*t* = 2 fs) and the five replicas with HMR (Δ*t* = 4 fs), shown in Figure S23, demonstrates that using TAMD/OTFP and HMR does not affect the stability of the MD integrator.

#### Translocation of a Single Sodium Ion

For each of the DEK^+1^A, DEK^0^A, DEE^–1^A, and DEE^0^A Na_v_1.2 systems, we ran six independent OTFP_1_ calculations, three starting from the homology model (Figure S15) and three starting from the cryo-EM structure (Figure S16). Then we calculated an average map by taking, for each point in CV space where the FESs are defined, the average of their FE values. The resulting averaged maps are reported in Figure 7, along with representative snapshots for the metastable and other states. Interestingly, the DEK^+1^A system shows the broader low-energy region, where it is possible to identify a gradually decreasing energy path for the ion through the SF and different configurations for the side chain of K1422. Conversely, all other systems display a single isolated minimum, each one associated with a unique orientation of the side chain of residue 1422.

For the DEK^+1^A system, the positions of the single Na^+^ and K1422 at the energy minima are well-correlated with the high-probability locations obtained from our standard MD simulations (see Figure 6) and with what is described in ref (93). In these configurations (see the states y_1_ and y_2_ in Figure 7), the Na^+^ is located at the inner portion of the SF (*r*_*z*_(Na_1_) ≈ 4 Å), in the proximity of the internal carbonyl oxygen atoms belonging to the two residues underneath the DEKA ring. The K1422 ammonium group is found either at the level of the DEKA backbone (at *r*_*z*_(K-NH_3_^+^) ≈ 11 Å, y_1_) or at a lower position (*r*_*z*_(K-NH_3_^+^) ≈ 8 Å, y_2_).

**Figure 8:**
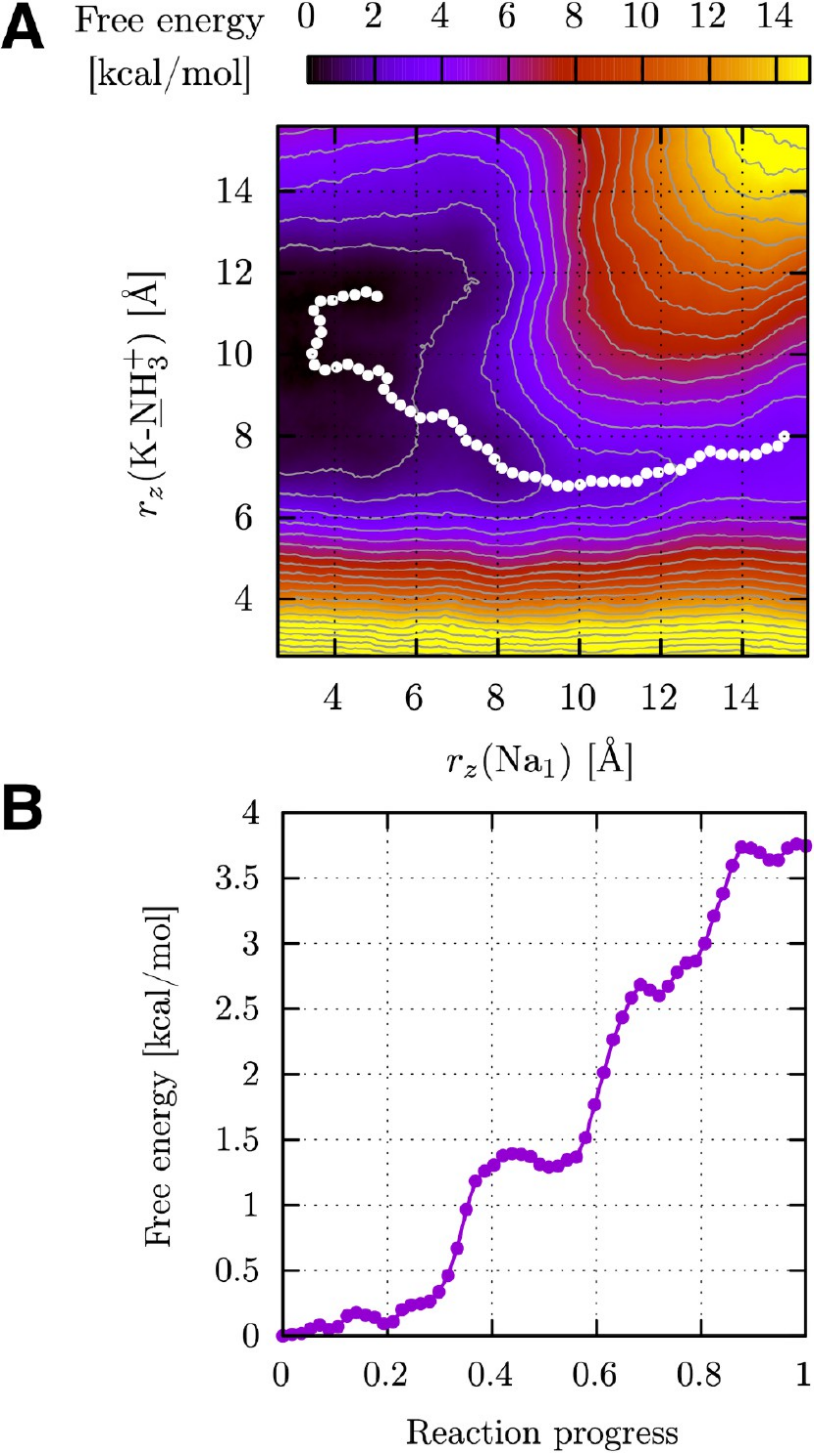
(A) MFEP (white dots) of the DEK^+1^A system’s averaged FES, superimposed on the corresponding map. (B) FE profile along the MFEP.

Using the string method,^137−139^ we computed the minimum free energy path (MFEP) over the DEK^+1^A surface, shown as a white trace over the corresponding map in Figure 8, where we also report the FE profile along the path. A video with snapshots of the representative configuration and volume occupancies along the entire MFEP is given in the Supporting Information. The MFEP allows us to reconstruct the following mechanism for single ion permeation: Na^+^ access to the upper part of the C/SF is possible only when K1422 is in a lower position, interacting with the upper ring of the internal carbonyls (configurations y_4_ and y_3_ in Figure 7); during ion permeation through the filter (*r*_*z*_(Na_1_) from 15 to 5 Å), the K1422 side chain switches from the downward to the upward conformation. When K1422 starts moving, the ion crosses it and reaches lower positions, making space for the side chain to move up. Once this is in the upward configuration, the ion finally adjusts to a slightly higher position. In terms of FE values, this last part of the path is essentially a descent toward the minimum. This picture is consistent with the role of the protonated K1422 suggested in refs (70) and (146), where it is proposed that in the up position the charged lysine interacts with the negatively charged side chain of the DEKA ring to block the access of ions from the extracellular space.

Opposite to the DEK^+1^A configuration, all the other systems’s FESs show an isolated minimum, again with the ion located in the region surrounded by the carbonyl oxygen atoms below the DE*A ring (where * indicates the variable residue among the different configurations), at 4 Å < *r*_*z*_(Na_1_) < 8 Å. In all three cases, the modification of the K1422 residue results in higher energy values in the upper part of the C/SF. The system with the DEK^0^A ring is characterized by a single metastable state in the FES, y_5_ (Figure 7), defined by the Na^+^ ion at *r*_*z*_(Na_1_) ≈ 6 Å and the *r*_*z*_(K-NH_2_) at ∼7 Å. Interestingly, other works investigated the role of different protonation states of the DEKA lysine^46,70^ in ionic permeation. While extensive unbiased MD simulations of a chimeric bacterial/eukaryotic channel^46^ suggested that the uncharged lysine plays a minor role in the selectivity process, Monte Carlo simulations on multiple eukaryotic structures^70^ proposed that the uncharged lysine is responsible for escorting the Na^+^ ion to the inner carbonyl rings, resulting in a configuration that is very similar to the one identified in our DEK^0^A TAMD/OTFP simulations (state y_5_). Also the K1422E mutation (either DEE^–1^A or DEE^0^A) affects the thermodynamic features of Na^+^ translocation. In the DEE^–1^A minimum (y_6_), the Na^+^ ion is localized at *r*_*z*_(Na_1_) ≈ 8 Å, with the E1422 side chain confined in the upper region of the SF, at *r*_*z*_(E-COO^–^) ≈ 14 Å. In the case of DEE^0^A, the FE minimum (y_7_) is located at *r*_*z*_(Na_1_) ≈ 7 Å and *r*_*z*_(E-COOH) ≈ 9 Å, i.e., with the E1422 CD atom in the proximity of the inner ring’s backbone, in a configuration similar to that of the DEK^0^A system (y_5_) but with the ion and the glutamate side chain in a slightly higher position.

#### Translocation of Two Sodium Ions

We then computed 2D FESs using the OTFP_2_ setup of CVs illustrated in Materials and Methods, i.e., by considering as CVs the vertical positions of the COM of the two ions and of the side chain of residue 1422. Indeed, multiple Na^+^ ion configurations were observed in a chimeric bacterial/mammalian model including the Na_v_1.2 filter^46^ and extensively discussed in many works presenting experimental results.^79−82^

**Figure 9:**
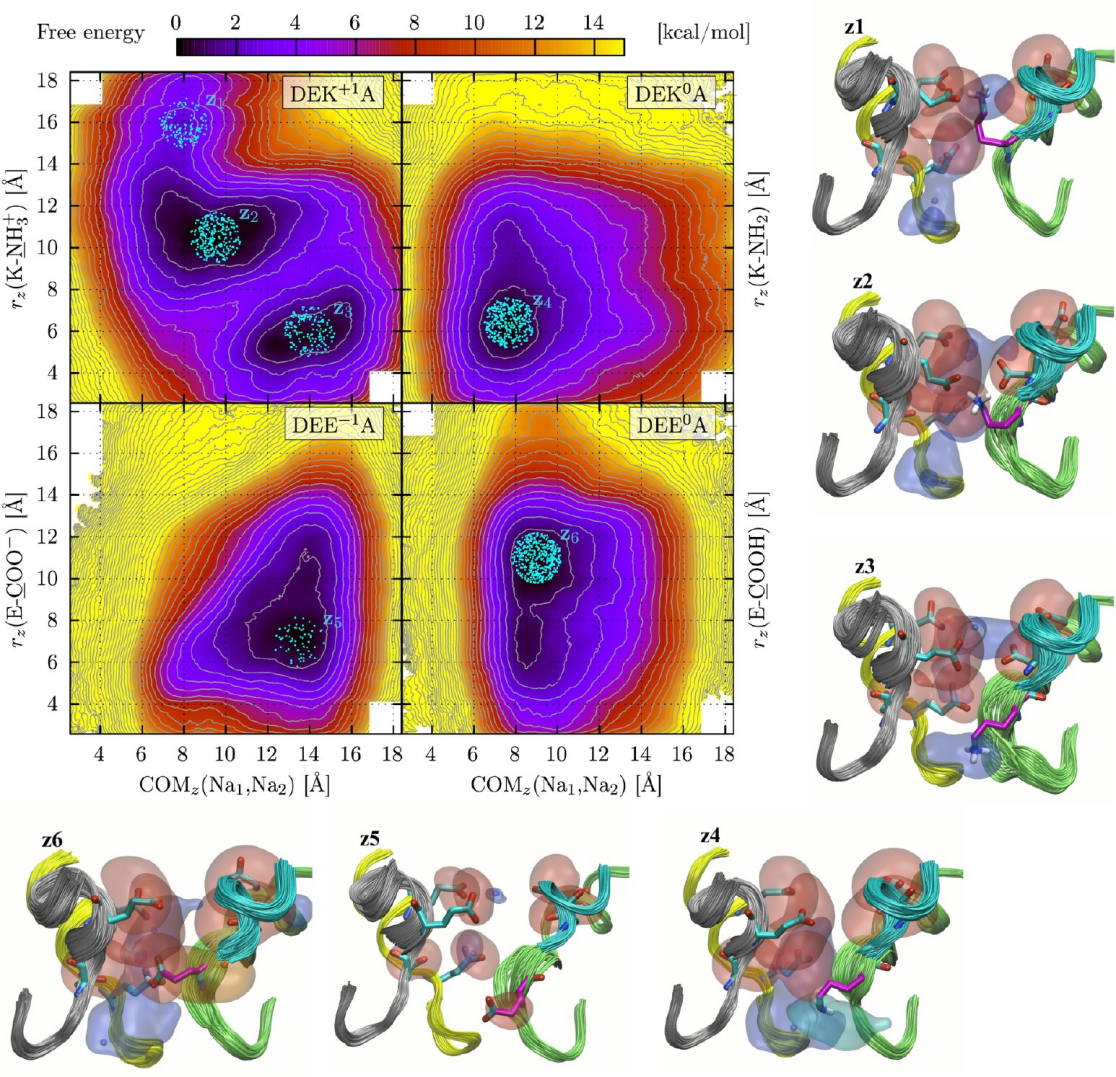
FESs for translocation of two Na^+^ ions in the SF of each of the four systems, as indicated in the labels (DEK^+1^A, DEK^0^A, DEE^–1^A, DEE^0^A). Cyan dots indicate the snapshots used for the representative configurations z_1_ to z_6_, which are reported as explained in Figure 7.

As detailed in Table S6, and similarly to what was described for the OTFP_1_ simulations, for each (DEK^+1^A, DEK^0^A, DEE^–1^A, DEE^0^A) system we performed six independent OTFP_2_ calculations, three using the homology model (Figure S17) and three the cryo-EM structure (Figure S18), and averaged the results in a single map. The final averaged maps are reported in Figure 9, along with a set of representative snapshots for the metastable and other states. The DEK^+1^A FES shows a shallow minimum (z_1_) and two deep FE minima (z_2_ and z_3_). The z_1_ configuration is characterized by the two ions at the inner position, with their COM_*z*_ at ∼8 Å and the lysine in proximity of the EEDD ring, nearly at the upper limit of the sampled space, *r*_*z*_(K-NH_3_^+^) ≈ 16 Å. The z_2_ and z_3_ states correspond to higher positions for the COM_*z*_ of the ions and lower positions for K1422, i.e., COM_*z*_(Na_1_, Na_2_) ≈ 10 Å and *r*_*z*_(K-NH_3_^+^) ≈ 11 Å for z_2_ and COM_*z*_(Na_1_, Na_2_) ≈ 14 Å and *r*_*z*_(K-NH_3_^+^) ≈ 6 Å for z_3_. Hence, by considering the sequence of states in the order z_3_ to z_1_, we can describe the movement of two Na^+^ ions within the C/SF as follows:

- z_3_: the two ions are at their highest positions, while K1422 is at its lowest, and the sequence of positive charges from the extracellular side to the inner part of the filter is [Na^+^, Na^+^, K1422-NH_3_^+^].
- z_2_: the ions move to a lower position (in particular the inner one), and K1422 switches to an intermediate one between them; the sequence of positive charges from the extracellular side to the inner region is now [Na^+^, K1422-NH_3_^+^, Na^+^].
- z_1_: from the previous state, a less stable configuration is accessible, characterized by a slightly lower position of the ions and a considerably higher location of K1422; the sequence of positive charges is now [K1422-NH_3_^+^, Na^+^, Na^+^].

These multiple features are completely lost in the maps of the other three systems DEK^0^A, DEE^–1^A, and DEE^0^A. The DEK^0^A FES shows a single minimum, z_4_, defined by COM_*z*_(Na_1_, Na_2_) ≈ 8 Å and *r*_*z*_(K-NH_2_) ≈ 6 Å, i.e., with the ions and the uncharged lysine at lower positions. This configuration of the side chain is again similar to that observed in ref (70).

When the DEKA filter is replaced by DEE^–1^A, the two ions are substantially trapped in the upper part of the SF (state z_5_, COM_*z*_(Na_1_, Na_2_) at ∼14 Å with one ion at the level of the EEDD motif and one ion in proximity of the DEEA ring), and their access to the inner region is precluded. In the case of the DEE^0^A system, the main metastable configuration (z_6_) corresponds to COM_*z*_(Na_1_, Na_2_) ≈ 9 Å and *r*_*z*_(E-COOH) ≈ 11 Å, with a less populated state with the uncharged glutamate at a lower position. This latter state is similar to z_4_, revealing again similarities among the two uncharged systems (DEK^0^A and DEE^0^A).

## Conclusions

Voltage-gated sodium (Na_v_) channels play a pivotal role in the regulation of excitable cells, and mutations in genes encoding them have been linked to numerous disorders affecting nervous system function, heart rhythm, and muscle contraction. Therefore, understanding the structural and mechanistic features of the cationic fluxes through the Na_v_ conductivity/selectivity filter (C/SF) is essential to accelerate the development of new efficient pharmacological treatments. In this context, a challenging but essential endeavor is to investigate the peculiar structural features of eukaryotic channels that are not obtainable from studying the high-resolution crystal structures of bacterial channels.

In this study, we used a combination of molecular modeling, standard MD, and enhanced sampling to provide a description of ion permeation through the SF of human neuronal Na_v_ channels. We combined results from MD simulations of homology-based models of Na_v_1.1, -1.2, and -1.6 and the recent cryo-EM Na_v_1.2 structure (PDB ID 6J8E) to describe the thermodynamics of Na^+^ residency within the C/SF. We ran simulations on multiple variants of the key SF DEKA ring by modifying the positively charged K (K1422 in Na_v_1.2), which is demonstrated to be essential for selectivity. Our simulations include a set of enhanced-sampling MD simulations employing the TAMD/OTFP method to reconstruct 2D FESs for single and double Na^+^ ions through the C/SF of the Na_v_1.2 structures. TAMD/OTFP has been previously assessed as an efficient tool to investigate systems with hidden FE barriers. FESs were obtained separately for two CV sets, the vertical position of one or two Na^+^ ions in the C/SF and that of the side chain of residue 1422. We also investigated the role of K1422 protonation and of charged and uncharged K1422E mutants. Moreover, the obtained FESs show that in the WT DEK^+1^A system there is a pathway through the C/SF with low FE values that the ions can follow to reach the inner portion of the filter, both as single- or double-ion configurations. We then described a putative mechanism of Na^+^ permeation in the case of the single-ion translocation using the string method. For all other systems (DEK^0^A, DEE^0^A, DEE^–1^A), the FESs display single isolated minima, mostly localized in the inner portion of the SF. Our simulations constitute a first attempt to investigate Na^+^ permeation in neuronal Na_v_ channels employing mammalian cryo-EM structures. They clearly show the metastable sites for Na^+^ in the C/SF and the process by which they are accessed. Moreover, they reveal the role of the lysine residue in controlling Na^+^ translocation, in one- and two-ion occupancy states. This was investigated in previous work,^46^ albeit using a modified structure of a bacterial Na_v_ channel where residues of the human Na_v_1.2 filter were grafted.

The present approach, leveraging the TAMD/OTFP methodology, may be used in further works to extend the space of sampling of the simulations and, in addition, to explore the selectivity mechanisms of neuronal Na_v_ channels.

The use of cryo-EM structures in atomistic simulations requires caution, mostly because of the different resolution observed in the various protein domains. Even when good local resolution is available (as for the hNa_v_1.4), the less resolved components of the structure can affect the fine balance between the residues lining the C/SF domain and the permeating ions. For this reason, during the simulations, it is important to monitor the structural stability and possible divergences from the initial configuration for the whole protein and for specific functionally relevant domains, as we reported here in detail. The definitive validation of cryo-EM structures and their characterization from a functional point of view remain long-term goals yet to be achieved. A significant step in this direction is represented by the recent boost of deep-learning-based approaches to predict protein structures,^147,148^ as it has been observed that predicted models of Na_v_ channels are quite close to cryo-EM structures.^149^ However, limited differences have been pointed out in specific protein domains,^150^ and further experimental and computational studies are necessary to understand their effects.^151,152^

It is also important to comment on the use of homology/comparative modeling. In our case, the application of this strategy is supported by the high sequence identity between neuronal hNa_v_ channels and the hNa_v_1.4 homologue (∼85% to ∼91% in the TM region, including the conserved EEDD and DEKA motifs in the C/SF), appropriate for obtaining high-quality homology models at atomic resolution.^94,105^ Such high sequence identity among the nine isoforms of hNa_v_s has also functional consequences, and it is considered a cause for the limited selectivity of therapeutics that target these proteins, leading to various off-target side effects.^153,154^ As for the other protein domains, we employed a conservative approach and maintained the long extracellular loops that have been previously excluded in other studies.^92^ Our homology modeling relies on the use of state-of-the-art software for protein structure predictions, SWISS-MODEL,^108^ one of the most widely used and effective programs in the field. Moreover, the models were refined with FG-MD,^110^ a fragment-guided MD sampling developed by the I-TASSER community in order to improve the quality of multisubunit protein models. Finally, the recent cryo-EM Na_v_1.2 structure^99^ was also investigated, since it is the only experimental one available for the channel at the moment and is therefore of relevance to verify any conclusion drawn from homology models.

Another key issue in atomistic simulations is the choice of the force field (FF). We employed here the most recent version of the CHARMM FF (CHARMM36m^113−115^), which to the best of our knowledge is the most-used one by the ion channel community (including Na_v_s^46,85,89^) and membrane protein simulators.^103,105,155−158^ An interesting comparison between the effects of CHARMM and AMBER^159,160^ FFs was recently reported,^161,162^ showing marked differences in the relative probability of conductive/occluded configurations of the KcsA channel’s SF. These results leave open challenges in atomistic simulations, and the assessment of our results with other FFs will be the subject of future work.

## Data Availability

The additional movies listed in the Supporting Information are available free of charge at https://github.com/BeatriceCorradi/Nav_Channels_movies.git.

## Supporting Information Available

Representation of the Na_v_1.1 and Na_v_1.6 SFs; superposition between the cryo-EM Na_v_1.2 structure (PDB ID 6J8E) and the homology-based Na_v_1.2 model; backbone RMSD calculations of the standard MD simulations; cross distance analysis; individual FESs for OTFP_1_ and OTFP_2_ (homology-based Na_v_1.2 structure); individual FESs for OTFP_1_ and OTFP_2_ (cryo-EM Na_v_1.2 structure, PDB ID 6J8E); average OTFP_1_ and OTFP_2_ for the homology-based Na_v_1.2 model in the presence of the extended exclusion rectangle; and list of additional movies from standard MD simulations (PDF)
Video with snapshots of the representative configuration and volume occupancies along the entire MFEP (AVI)

## Supplementary Material

ct2c00990_si_001.pdf

ct2c00990_si_002.avi

## Abbreviations

CDIST: cross distance
C/SF: conductivity/selectivity filter
cryo-EM: cryogenic electron microscopy
DEKA: notation for the Asp, Glu, Lys, Ala residues in the inner DEKA ring of the selectivity filter
EEDD: notation for the Asp, Asp, Glu, Glu residues in the outer EEDD ring
FF: force field
FE: free energy
MD: molecular dynamics
Na_v_: voltage gated sodium channel
OTFP: on-the-fly free energy parametrization
PD: pore domain
POPC: 1-palmitoyl-2-oleoyl-*sn*-glycero-3-phosphocholine
RMSD: root-mean-square deviation
S1–S6: labels for the six α helices in each domain of a human voltage gated sodium Na_v_ channel
TAMD: temperature-accelerated molecular dynamics
SF: selectivity filter
