# Supplementary material for: Molecular Dynamics Simulations of Ion Permeation in Human Voltage-Gated Sodium Channels
Source: J Chem Theory Comput. 2023 Apr 28;19(10):2953–72. doi: 10.1021/acs.jctc.2c00990 (PMC10210251; doi:10.1021/acs.jctc.2c00990)
Supplement: Supplementary file 1 — ct2c00990_si_001.pdf [file ct2c00990_si_001.pdf]

Molecular Dynamics Simulations of Ion Permeation in human NaV Channels - Supplementary Information

Giulio Alberini [Δ,†,‡] Sergio Alexis Paz [Δ,¶,§] Beatrice Corradi,[†,‖] Cameron F. Abrams,[*,⊥] Fabio Benfenati,[*,†,‡] and Luca Maragliano[*,†,#]

†*Center for Synaptic Neuroscience and Technology (NSYN@UniGe), Istituto Italiano di Tecnologia, Largo Rosanna Benzi, 10, 16132, Genova, Italy.*

‡*IRCCS Ospedale Policlinico San Martino, Largo Rosanna Benzi, 10, 16132, Genova, Italy.*

¶*Universidad Nacional de Córdoba. Facultad de Ciencias Químicas. Departamento de Química Teórica y Computacional. Córdoba (X5000HUA), Argentina.*

§*Consejo Nacional de Investigaciones Científicas y Técnicas (CONICET), Instituto de Fisicoquímica de Córdoba (INFIQC), Córdoba (X5000HUA), Argentina.*

‖*Department of Experimental Medicine, Università degli Studi di Genova, Viale Benedetto XV, 3, 16132, Genova, Italy.*

⊥*Department of Chemical and Biological Engineering, Drexel University, Philadelphia, PA 19104, United States.*

#*Department of Life and Environmental Sciences, Polytechnic University of Marche, Via Brecce Bianche, 60131, Ancona, Italy.*

E-mail: cfa22@drexel.edu; fabio.benfenati@iit.it; l.maragliano@univpm.it

[Δ] Giulio Alberini and S. Alexis Paz should be regarded as joint first authors.

Definition of the Cross Distances

In this section, we report the list of the cross distances (CDISTs) mapped during the standard MD simulations. The average for each CDIST is reported in **Table S3**.

Here, atoms are defined in the CHARMM notation, with the following assumptions:

- CA defines the C_α atoms of the backbone for each residue;
- all the others are the most external carbon atoms in the side-chain of each selected residue.

1. EEDD motif

    - **d1A**: E387CA - D1426CA; **d1B**: E387CD - D1426CG
    - **d2A**: E945CA - D1717CA; **d2B**: E945CD - D1717CG

Table S1: Residue numbering in the EEDD domain (d1-d2).

System	Rep. DI	Rep. DII	Rep. DIII	Rep. DIV
NaV1.4	E409	E764	D1248	D1539
NaV1.1	E385	E954	D1436	D1727
**NaV1.2**	**E387**	**E945**	**D1426**	**D1717**
NaV1.6	E 373	E939	D1417	D1708

2. DEKA motif

    - **d3A** D384CA - K1422CA; **d3B** D384CG - K1422CE
    - **d4A** E942CA - A1714CA; **d4B** E942CD - A1714CB

Table S2: Residue numbering in the DEKA domain (d3-d4).

System	Rep. DI	Rep. DII	Rep. DIII	Rep. DIV
NaV1.4	D406	E761	K1244	A1536
NaV1.1	D382	E951	K1432	A1724
**NaV1.2**	**D384**	**E942**	**K1422**	**A1714**
NaV1.6	D370	E936	K1413	A1705

3. BACKBONE scaffold

- **d5A** COM between T382CA and Q383CA - COM between T1420CA and F1421CA; **d5B** COM between T382O and Q38O - COM between T1420O and F1421O
- **d6A** COM between C940CA and G941CA - COM T1712CA and S1713CA; **d6B** COM between C940O and G941O - COM T1712O and S1713O

Table S3: Residue numbering (d5-d6).

System	Rep. DI	Rep. DII	Rep. DIII	Rep. DIV
NaV1.4	T404-Q405	C759-G760	T1242-F1243	T1534-S1535
NaV1.1	T380-Q381	C949-G950	T1430-F1431	T1722-S1723
**NaV1.2**	**T382-Q383**	**C940-G941**	**T1420-F1421**	**T1712-S1713**
NaV1.6	T368-Q369	C934-G935	T1411-F1412	T1703-S1704

- **d7A** L421CA-I1469CA; **d7B** L421CG-I1469CB
- **d8A** F978CA - I1775CA; **d8B** COM among the carbon atoms of the F978 aromatic ring - I1775CB
- **d9A** F978CA - Y1771CA; **d9B** COM among the carbon atoms of the F978 aromatic ring - COM among the carbon atoms of the Y1771 aromatic ring

Table S4: Residue numbering (d7-d8-d9).

System	Rep. DI	Rep. DII	Rep. DIII	Rep. DIV
NaV1.4	L443	F797	I1291	I1597-Y1593
NaV1.1	L419	F987	I1479	I1785-Y1781
**NaV1.2**	**L421**	**F978**	**I1469**	**I1775-Y1771**
NaV1.6	L407	F972	I1460	I1765-Y1761

Additional Supplementary tables

Table S5: Summary of the standard MD simulations with a 150 mM NaCl concentration.

**Na_v (C/SF)**	**Configuration**	**System**	**Method**	**Δt**	**Time**
1.2 (DE$\mathbf{K}^{+1}$A)	VSDs+Pore	Model	MD	2 fs	500 ns × 1
1.1 (DE$\mathbf{K}^{+1}$A)	VSDs+Pore	Model	MD	4 fs	500 ns × 1
1.2 (DE$\mathbf{K}^{+1}$A)	VSDs+Pore	Model	MD	4 fs	500 ns × 1
1.6 (DE$\mathbf{K}^{+1}$A)	VSDs+Pore	Model	MD	4 fs	500 ns × 1
1.2 (DE$\mathbf{K}^{+1}$A)	VSDs+Pore	Cryo-EM	MD	4 fs	500 ns × 2
1.2 (DE$\mathbf{K}^{+1}$A)	Pore	Cryo-EM	MD	4 fs	100 ns × 12
1.2 (DE$\mathbf{K}^{+1}$A)	Pore	Cryo-EM	MD	4 fs	∼ 190 ns

Table S6: Summary of the TAMD-OTFP simulations with a 150 mM NaCl concentration. (*) indicates simulations that were performed in bigger exclusion rectangle that have vertices (-6, -6, -4) and (6, 6, 22) Å instead of (-6, -6, -4) and (6, 6, 18) Å. (**) indicates simulations that were performed using HMR and the standard exclusion rectangle that have vertices (-6, -6, -4) and (6, 6, 18) Å.

**Na_v (SF)**	**K/E1422**	**Conf.**	**System**	**Method**	**Time length**	**Time step**
1.2 (DE$\mathbf{K}^{+1}$A)	**charged**	Pore	Model	$OTFP_1$	100 ns × 3	2 fs
1.2 (DE$\mathbf{K}^{+1}$A)	**charged**	Pore	Model	$OTFP_2$	100 ns × 3	2 fs
1.2 (DE$\mathbf{K}^{+1}$A)	**charged**	Pore	Model	$OTFP_1$	100 ns × 3	2 fs *
1.2 (DE$\mathbf{K}^{+1}$A)	**charged**	Pore	Model	$OTFP_2$	100 ns × 3	2 fs *
1.2 (DE$\mathbf{K}^{0}$A)	**uncharged**	Pore	Model	$OTFP_1$	100 ns × 3	2 fs
1.2 (DE$\mathbf{K}^{0}$A)	**uncharged**	Pore	Model	$OTFP_2$	100 ns × 3	2 fs
1.2 (DE$\mathbf{E}^{-1}$A)	**charged**	Pore	Model	$OTFP_1$	100 ns × 3	2 fs
1.2 (DE$\mathbf{E}^{-1}$A)	**charged**	Pore	Model	$OTFP_2$	100 ns × 3	2 fs
1.2 (DE$\mathbf{E}^{0}$A)	**uncharged**	Pore	Model	$OTFP_1$	100 ns × 3	2 fs
1.2 (DE$\mathbf{E}^{0}$A)	**uncharged**	Pore	Model	$OTFP_2$	100 ns × 3	2 fs
1.2 (DE$\mathbf{K}^{+1}$A)	**charged**	Pore	Cryo-EM	$OTFP_1$	∼ 150 ns × 3	2 fs
1.2 (DE$\mathbf{K}^{+1}$A)	**charged**	Pore	Cryo-EM	$OTFP_2$	∼ 150 ns × 3	2 fs
1.2 (DE$\mathbf{K}^{0}$A)	**uncharged**	Pore	Cryo-EM	$OTFP_1$	∼ 150 ns × 3	2 fs
1.2 (DE$\mathbf{K}^{0}$A)	**uncharged**	Pore	Cryo-EM	$OTFP_2$	∼ 150 ns × 3	2 fs
1.2 (DE$\mathbf{E}^{-1}$A)	**charged**	Pore	Cryo-EM	$OTFP_1$	∼ 125 ns × 3	2 fs
1.2 (DE$\mathbf{E}^{-1}$A)	**charged**	Pore	Cryo-EM	$OTFP_2$	∼ 125 ns × 3	2 fs
1.2 (DE$\mathbf{E}^{0}$A)	**uncharged**	Pore	Cryo-EM	$OTFP_1$	∼ 125 ns × 3	2 fs
1.2 (DE$\mathbf{E}^{0}$A)	**uncharged**	Pore	Cryo-EM	$OTFP_2$	∼ 150 ns × 3	2 fs
1.2 (DE$\mathbf{K}^{+1}$A)	**charged**	Pore	Cryo-EM	$OTFP_1$	∼ 300 ns × 5	4 fs **

Table S7: Summary of the cross distances measured in the Cryo-EM $Na_v1.2$ structure PDB: 6J8E (second column) and as the average among the mean values of the single standard MD simulations (third column).

Cross Distance	PDB ID:6J8E	$\mu \pm \sigma$ (Å)
d1A	17.9	17.06 ± 0.37
d1B	13.2	12.50 ± 0.40
d2A	17.6	19.37 ± 1.04
d2B	12.0	13.88 ± 1.01
d3A	10.8	10.45 ± 0.64
d3B	9.1	8.60 ± 0.57
d4A	11.9	12.13 ± 0.90
d4B	10.2	10.94 ± 0.81
d5A	11.3	12.93 ± 0.43
d5B	11.2	9.41 ± 0.35
d6A	11.6	14.21 ± 1.29
d6B	12.9	10.21 ± 0.62
d7A	13.7	13.30 ± 1.07
d7B	11.1	9.79 ± 1.01
d8A	15.7	15.94 ± 1.69
d8B	11.6	14.01 ± 2.03
d9A	15.7	14.11 ± 0.50
d9B	12.3	9.14 ± 0.43

Supplementary Figures

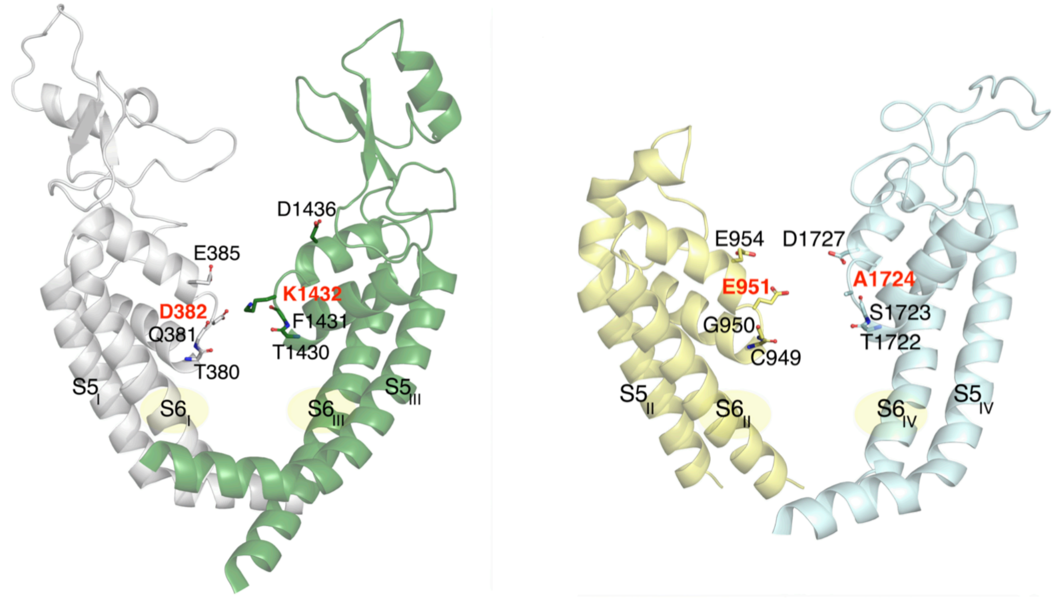

Figure S1: Representation of the asymmetric Na_v1.1 C/SF. The residues of the external $E_IE_{II}D_{III}D_{IV}$ motif, above the DEKA domain, are also shown.

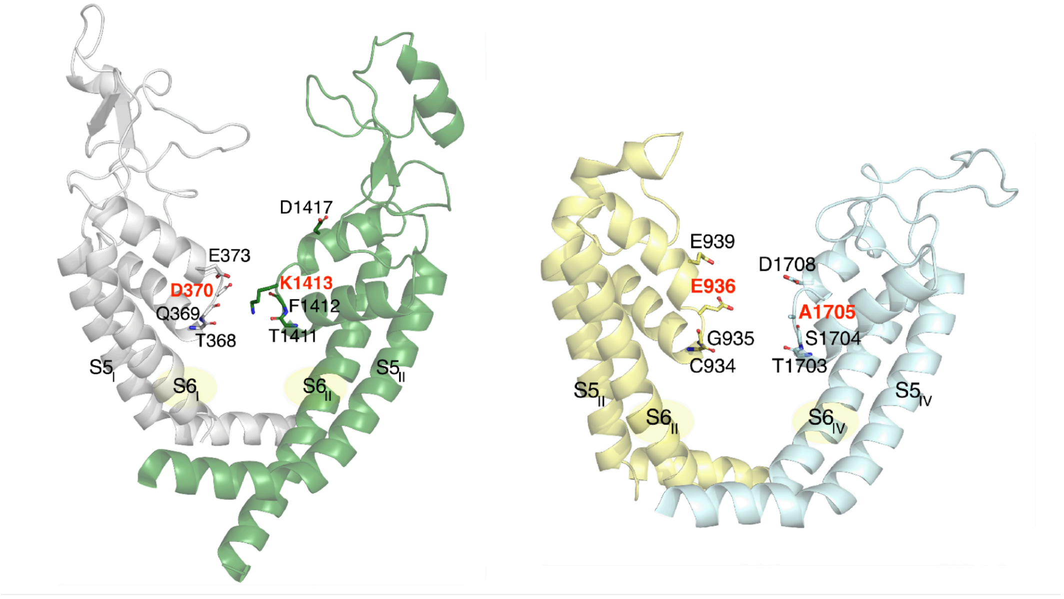

Figure S2: Representation of the asymmetric Na_v1.6 C/SF. The residues of the external $E_IE_{II}D_{III}D_{IV}$ motif, above the DEKA domain, are also shown.

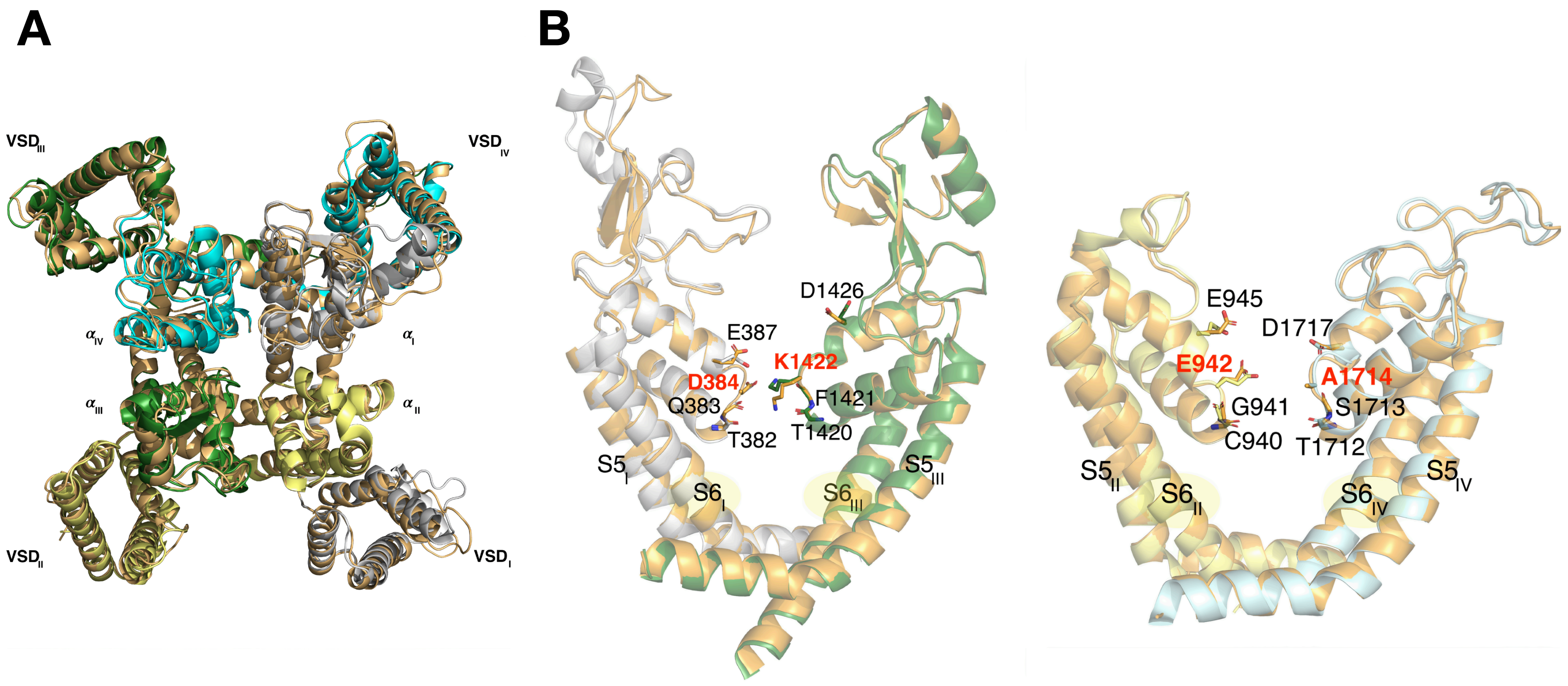

Figure S3: Superposition between the Cryo-EM Na$_v$1.2 structure (PDB ID: 6J8E), in brown, and the homology-based model of the same channel, represented with the same legend of Figure 1: domain DI, gray; domain DII, yellow; domain DIII, green; domain DIV, cyan. **A.** Extracellular view of the two superimposed structure. **B.** Representation of the asymmetric Na$_v$1.2 C/SF in the two configurations.

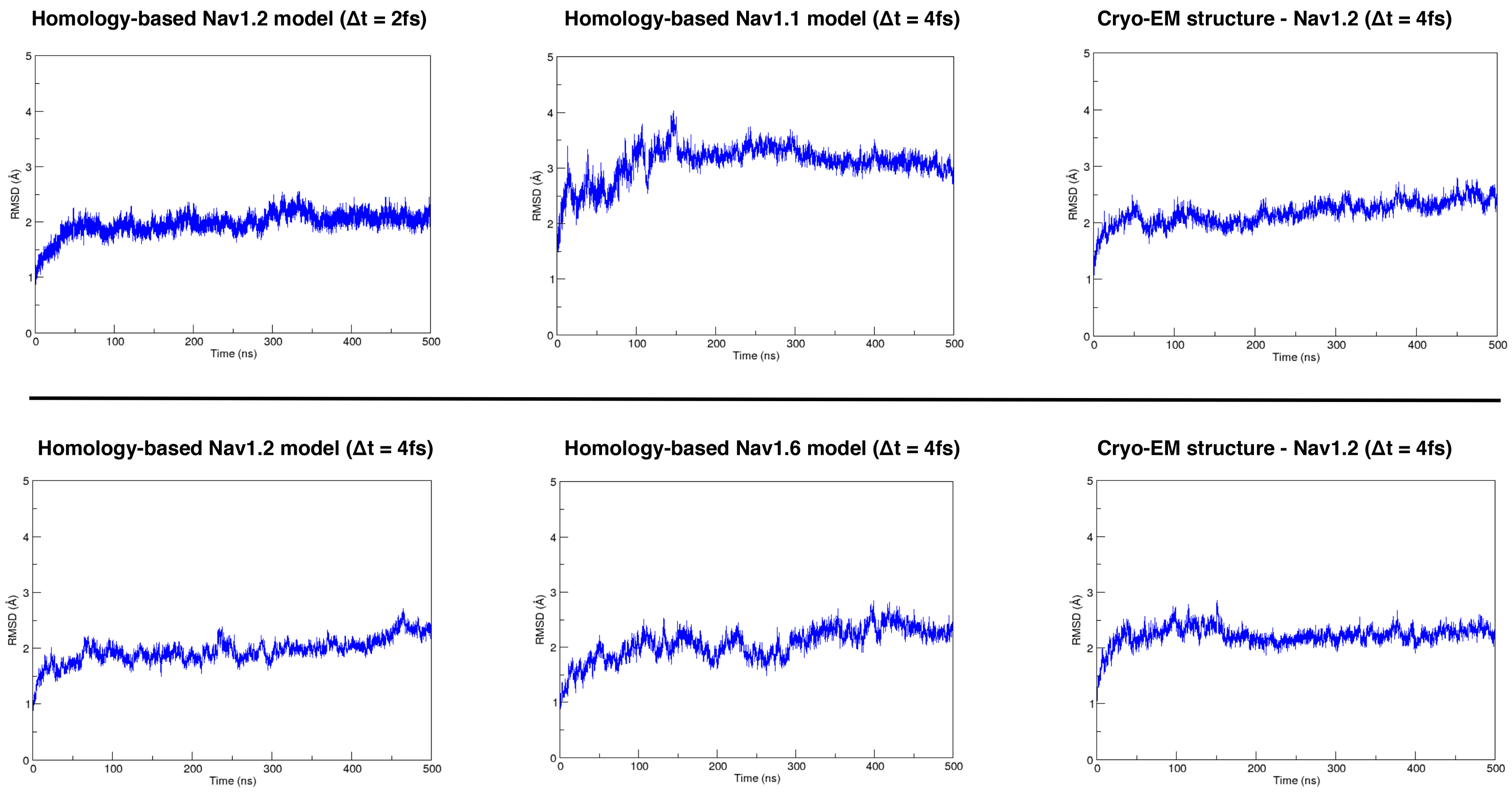

Figure S4: Backbone RMSD of the pore region (S5-S6 helices + P-loops) for each system during standard MD simulations.

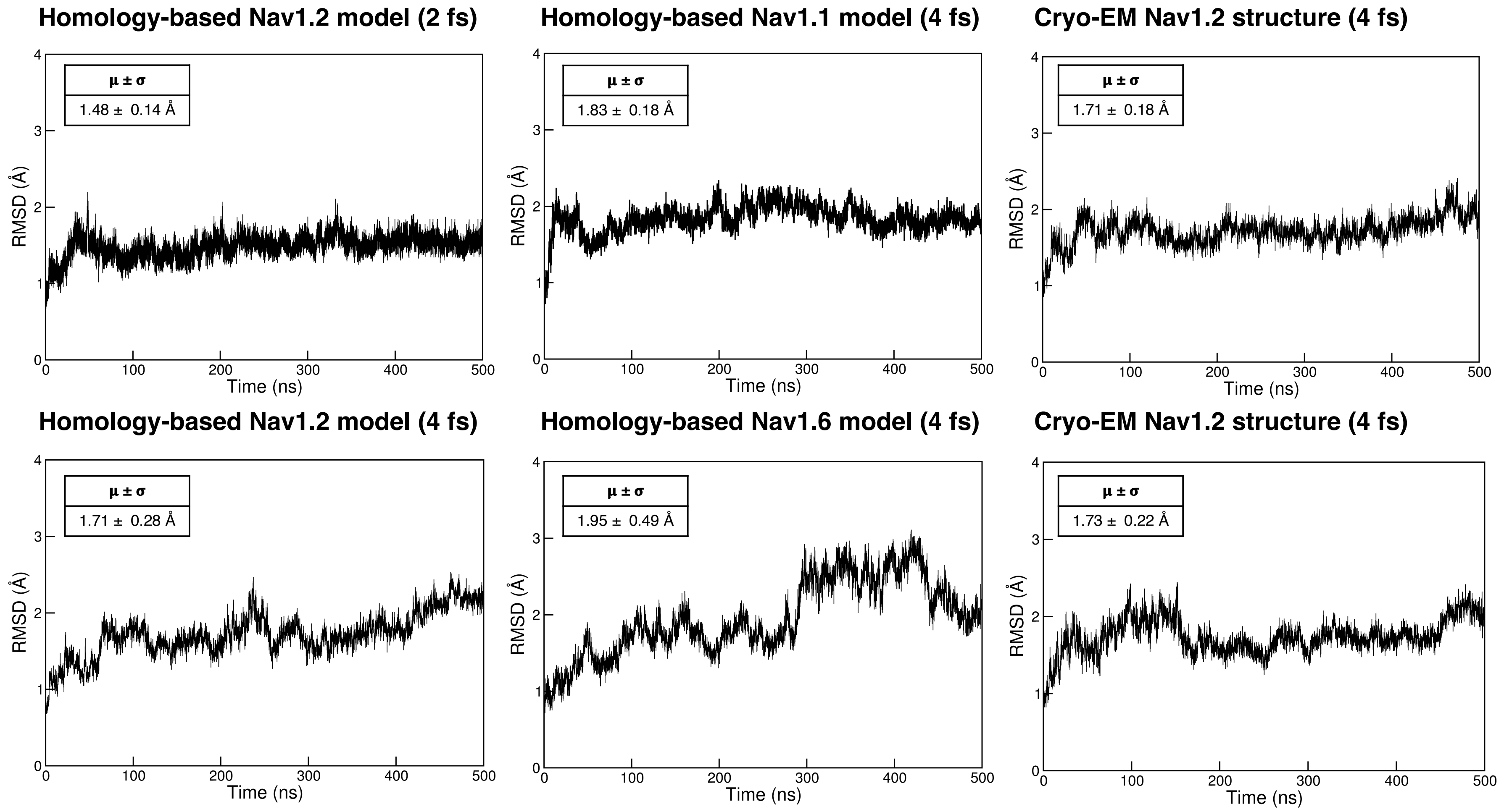

Figure S5: Backbone RMSD of the P-loops for each system during standard MD simulations. We also report the average (μ) and the standard deviation (σ) of the RMSD for each simulation.

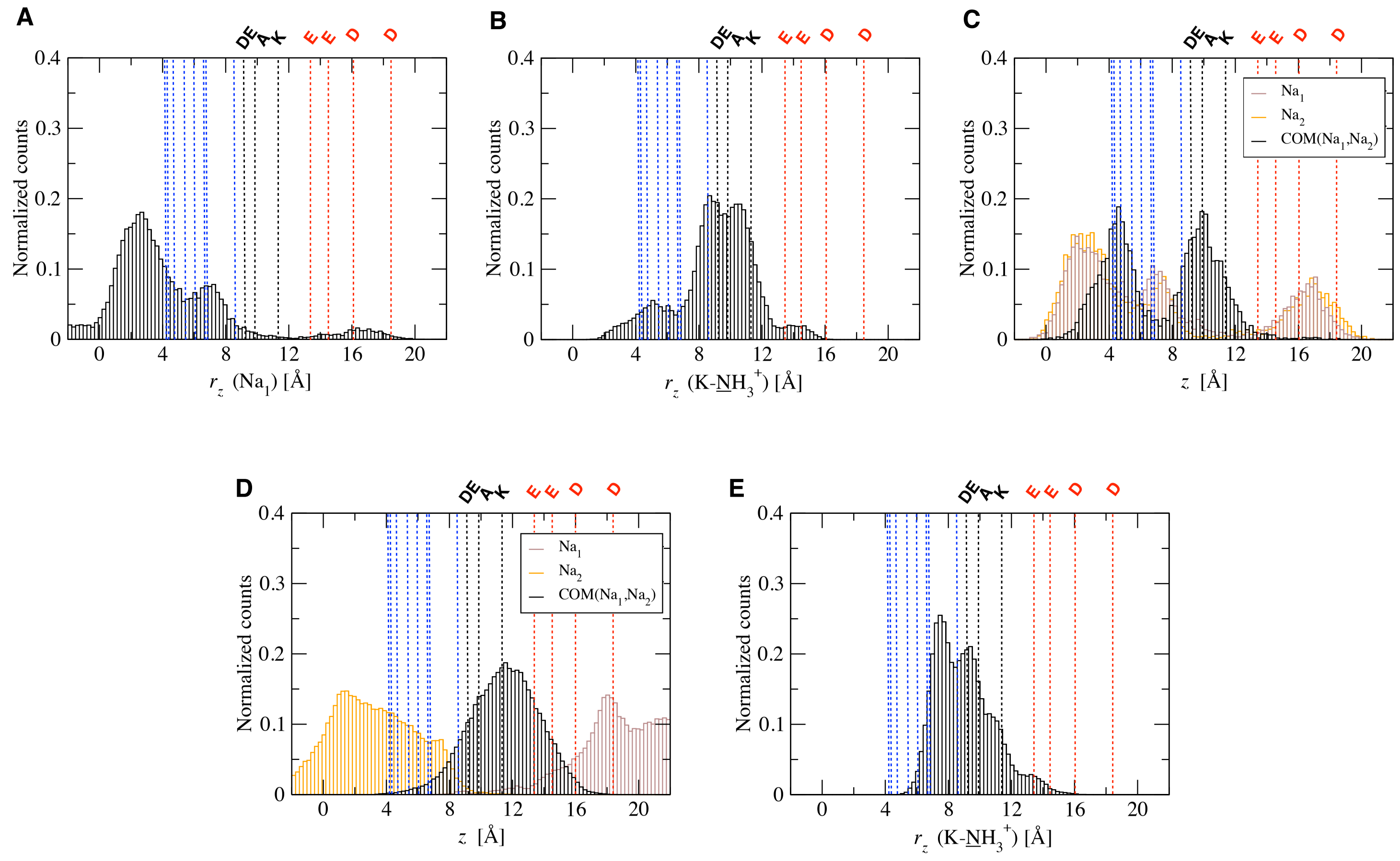

Figure S6: Histograms of the z-coordinate's values of (**A**) a single Na^+ ion in the C/SF; (**B**) the ammonium nitrogen of the DEKA ring; (**C**) two Na^+ ions and their COM in the double occupancy events; **D**, **E**, two Na^+ ions, their COM and the DEKA ammonium nitrogen in the additional simulations of the WT Cryo-EM $Na_v1.2$ structure (HMR set-up).

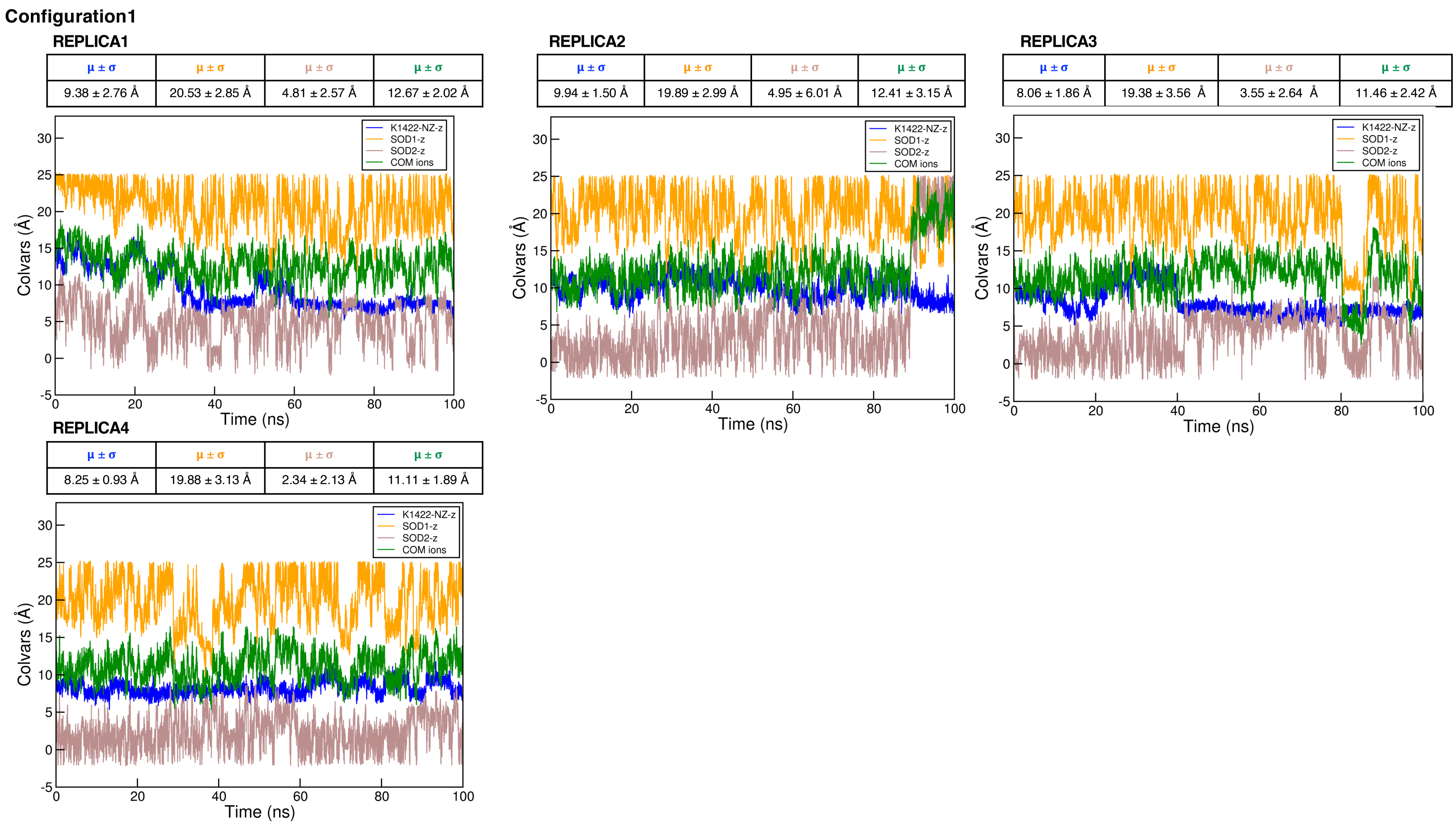

Figure S7: Additional MD of the WT Cryo-EM $Na_v1.2$ structure with two Na^+ confined in the C/SF (HMR set-up). Results from configuration 1. For each simulation, we report the z-coordinate of the two Na^+ ions in the C/SF, of the COM of the two cations and of the nitrogen atom of the K1422 side-chain.

Configuration1

REPLICA1

μ ± σ	μ ± σ	μ ± σ	μ ± σ
9.38 ± 2.76 Å	20.53 ± 2.85 Å	4.81 ± 2.57 Å	12.67 ± 2.02 Å

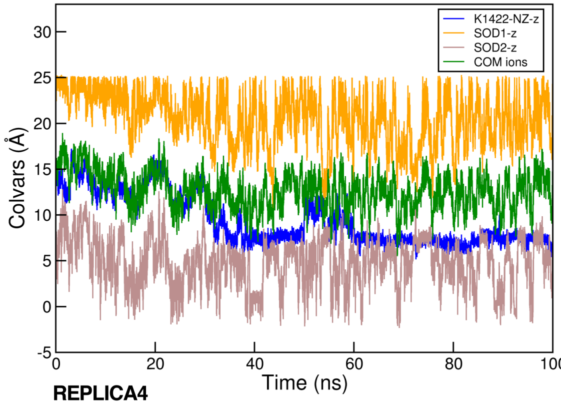

REPLICA2

μ ± σ	μ ± σ	μ ± σ	μ ± σ
9.94 ± 1.50 Å	19.89 ± 2.99 Å	4.95 ± 6.01 Å	12.41 ± 3.15 Å

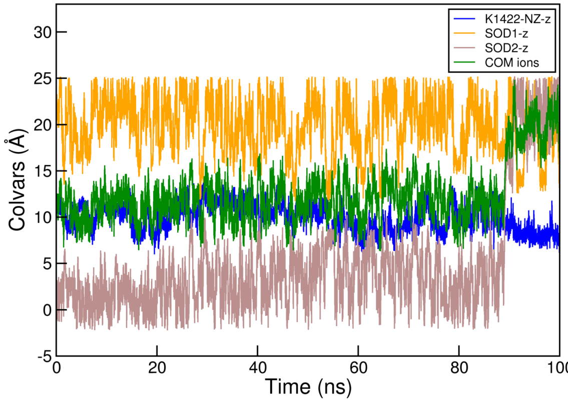

REPLICA3

μ ± σ	μ ± σ	μ ± σ	μ ± σ
8.06 ± 1.86 Å	19.38 ± 3.56 Å	3.55 ± 2.64 Å	11.46 ± 2.42 Å

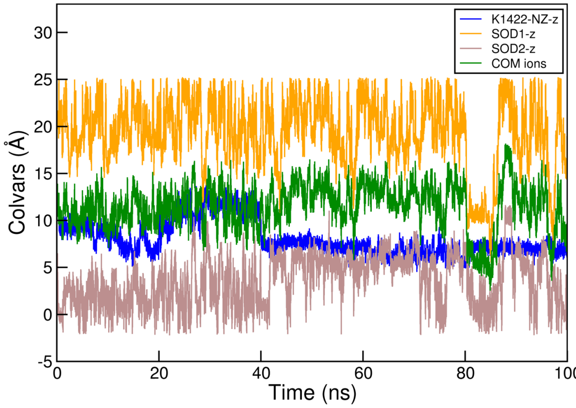

REPLICA4

μ ± σ	μ ± σ	μ ± σ	μ ± σ
8.25 ± 0.93 Å	19.88 ± 3.13 Å	2.34 ± 2.13 Å	11.11 ± 1.89 Å

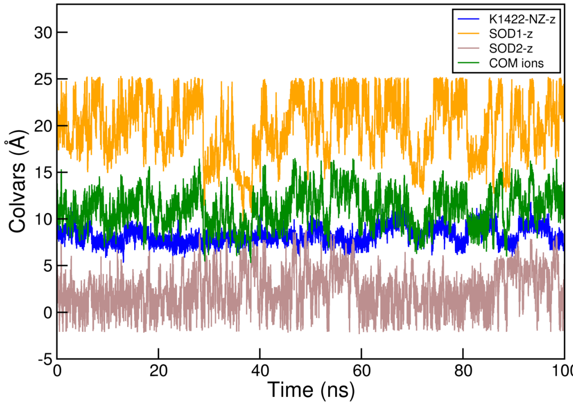

Figure S8: Additional MD of the WT Cryo-EM $Na_v1.2$ structure with two Na^+ confined in the C/SF (HMR set-up). Results from configuration 2. For each simulation, we report the z-coordinate of the two Na^+ ions in the C/SF, of the COM of the two cations and of the nitrogen atom of the K1422 side-chain.

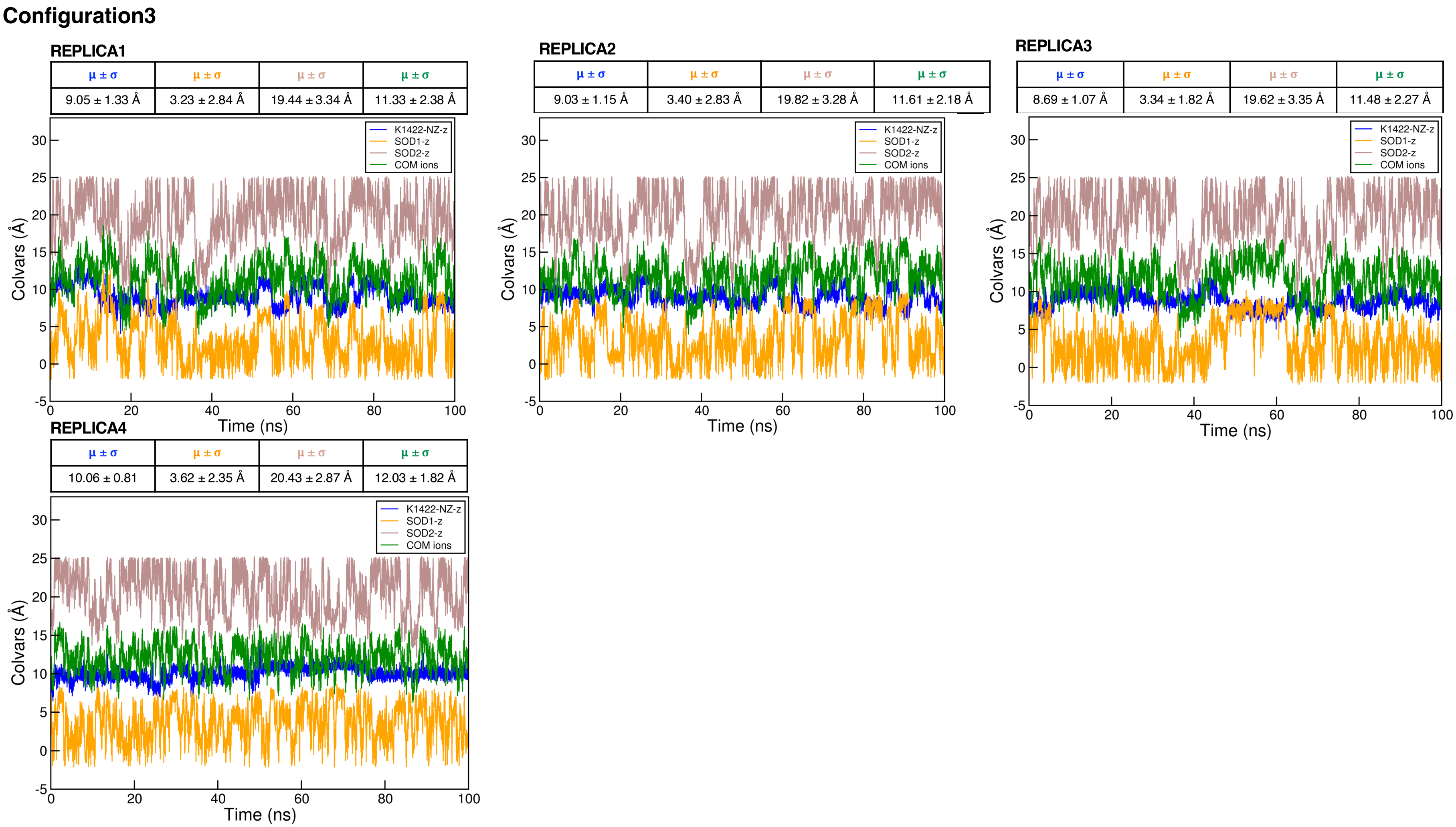

Figure S9: Additional MD of the WT Cryo-EM $Na_v1.2$ structure with two Na^+ confined in the C/SF (HMR set-up). Results from configuration 3. For each simulation, we report the z-coordinate of the two Na^+ ions in the C/SF, of the COM of the two cations and of the nitrogen atom of the K1422 side-chain.

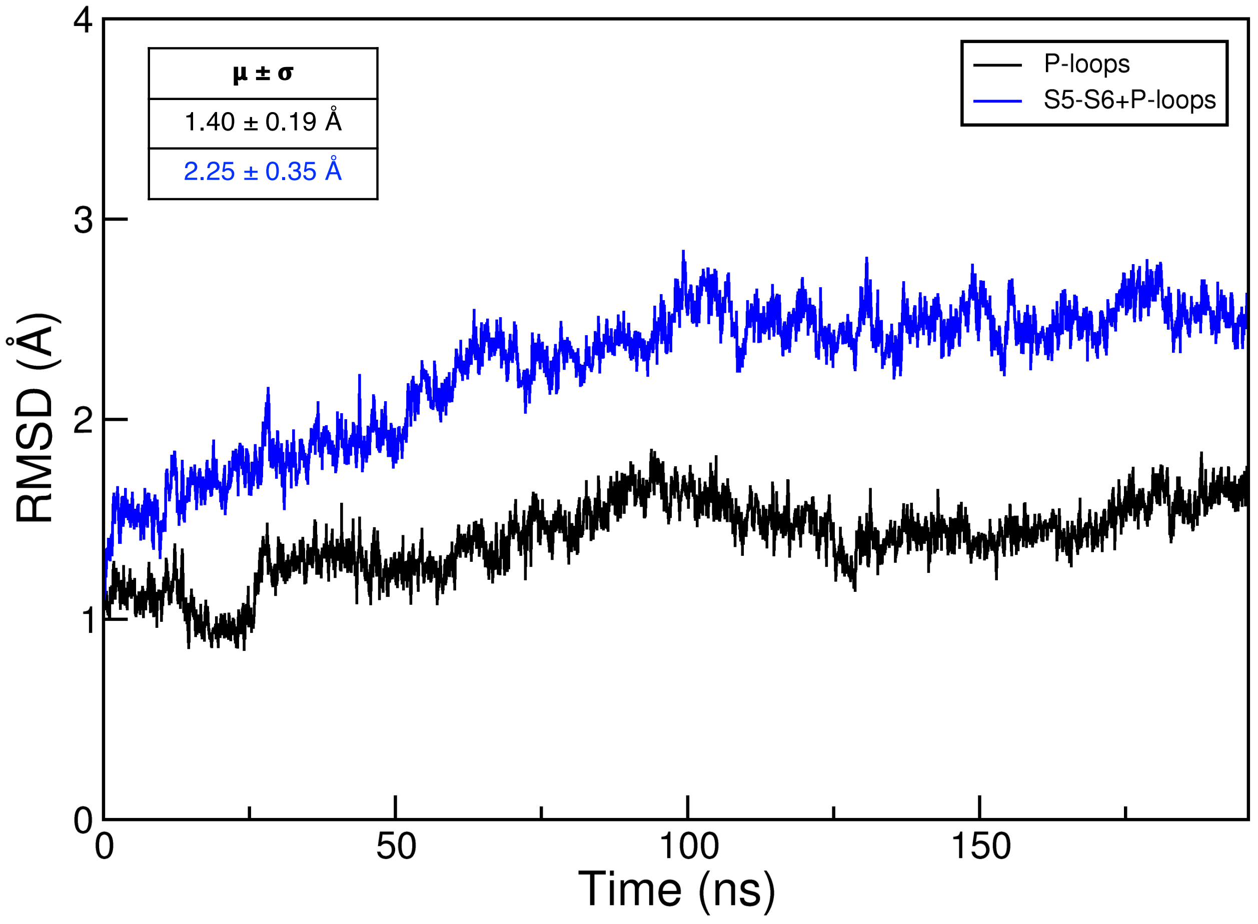

Figure S10: Backbone RMSDs for the pore only configuration from the Cryo-EM $Na_v1.2$ structure, PDB ID: 6J8E (black: P-loops and blue: S5-S6 helices + P-loops). For each calculation, we report the average (μ) and the standard deviation (σ).

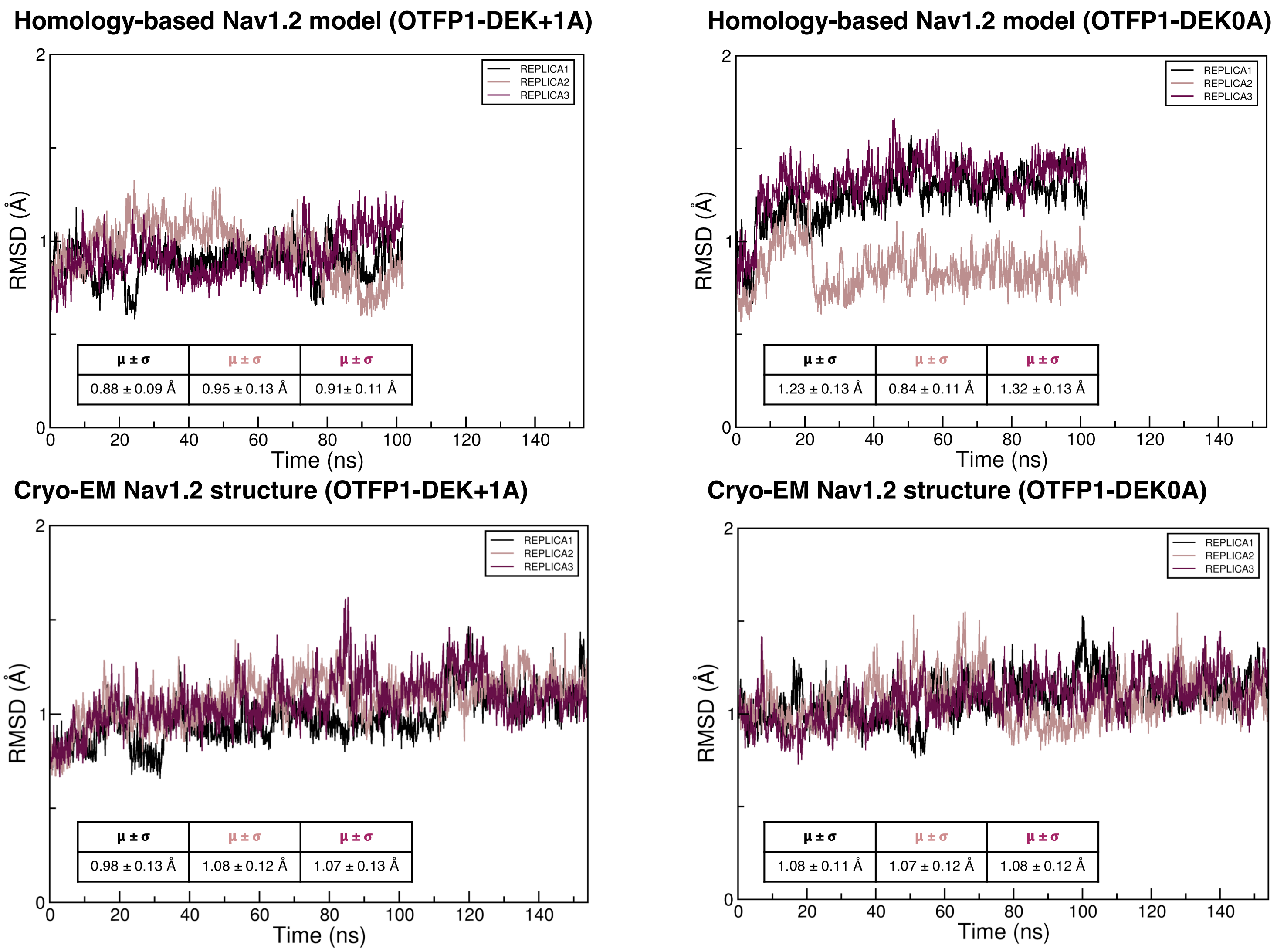

Figure S11: RMSD of the backbone P-loops (DEKA SF) for each system during the $OTFP_1$ simulations. We also report the average (μ) and the standard deviation (σ) of the RMSD. For each calculation, we report the average (μ and the standard deviation σ).

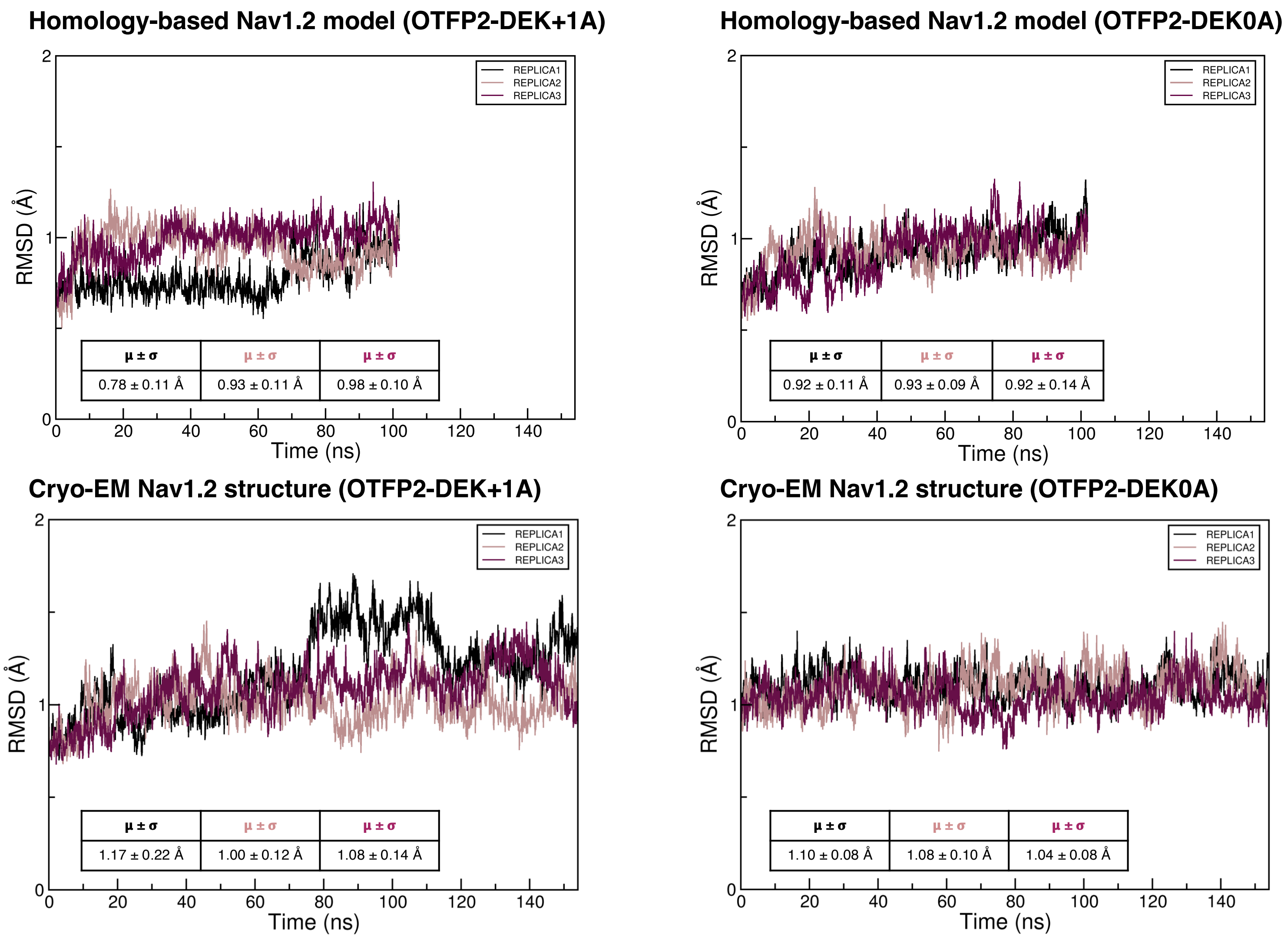

Figure S12: RMSD of the backbone P-loops (DEKA SF) for each system during the $OTFP_2$ simulations. We also report the average (μ) and the standard deviation (σ) of the RMSD. For each calculation, we report the average (μ and the standard deviation σ).

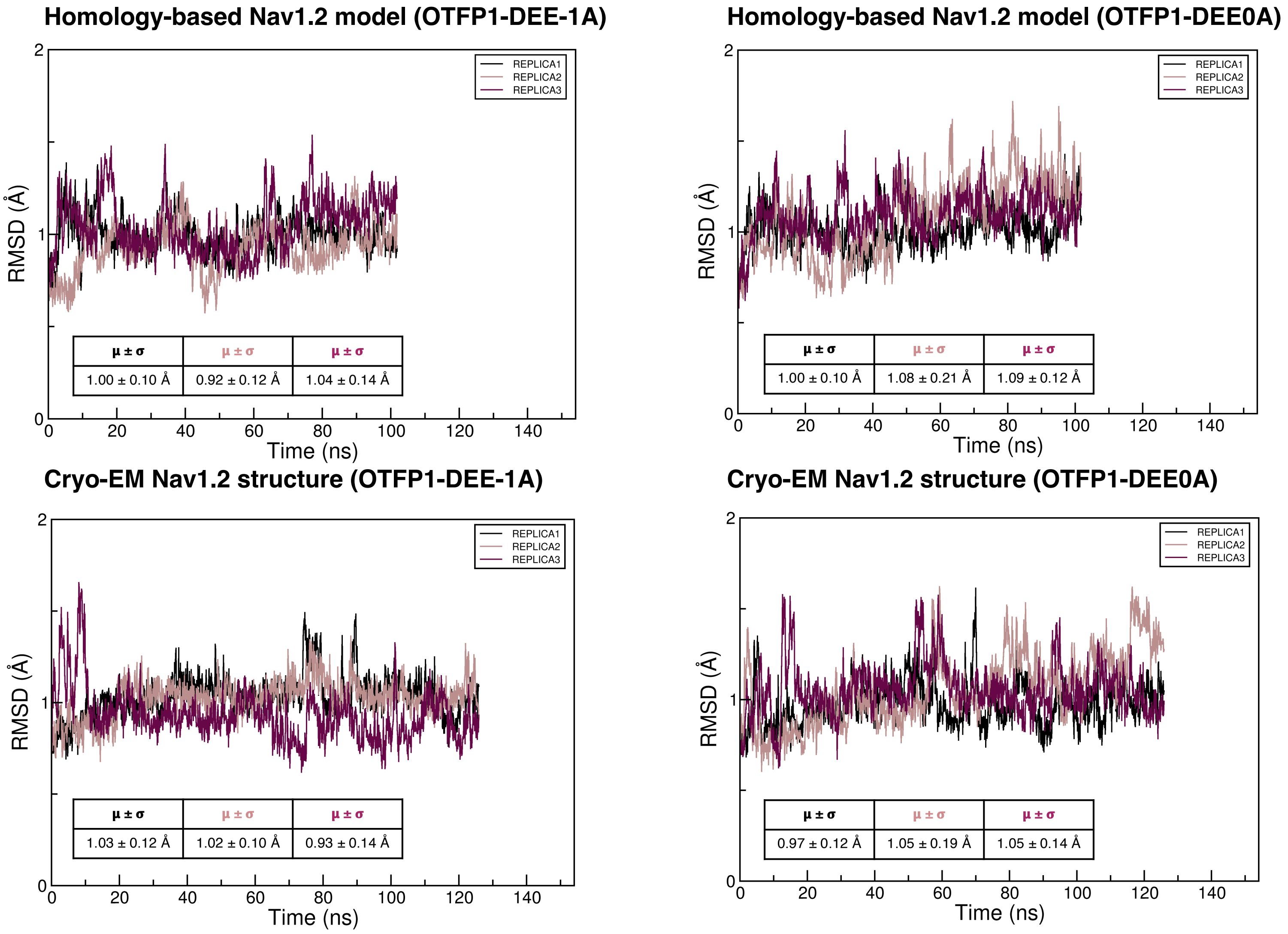

Figure S13: RMSD of the backbone P-loops of (DEEA SF) for each system during the $OTFP_1$ simulations. We also report the average (μ) and the standard deviation (σ) of the RMSD. For each calculation, we report the average (μ and the standard deviation σ)

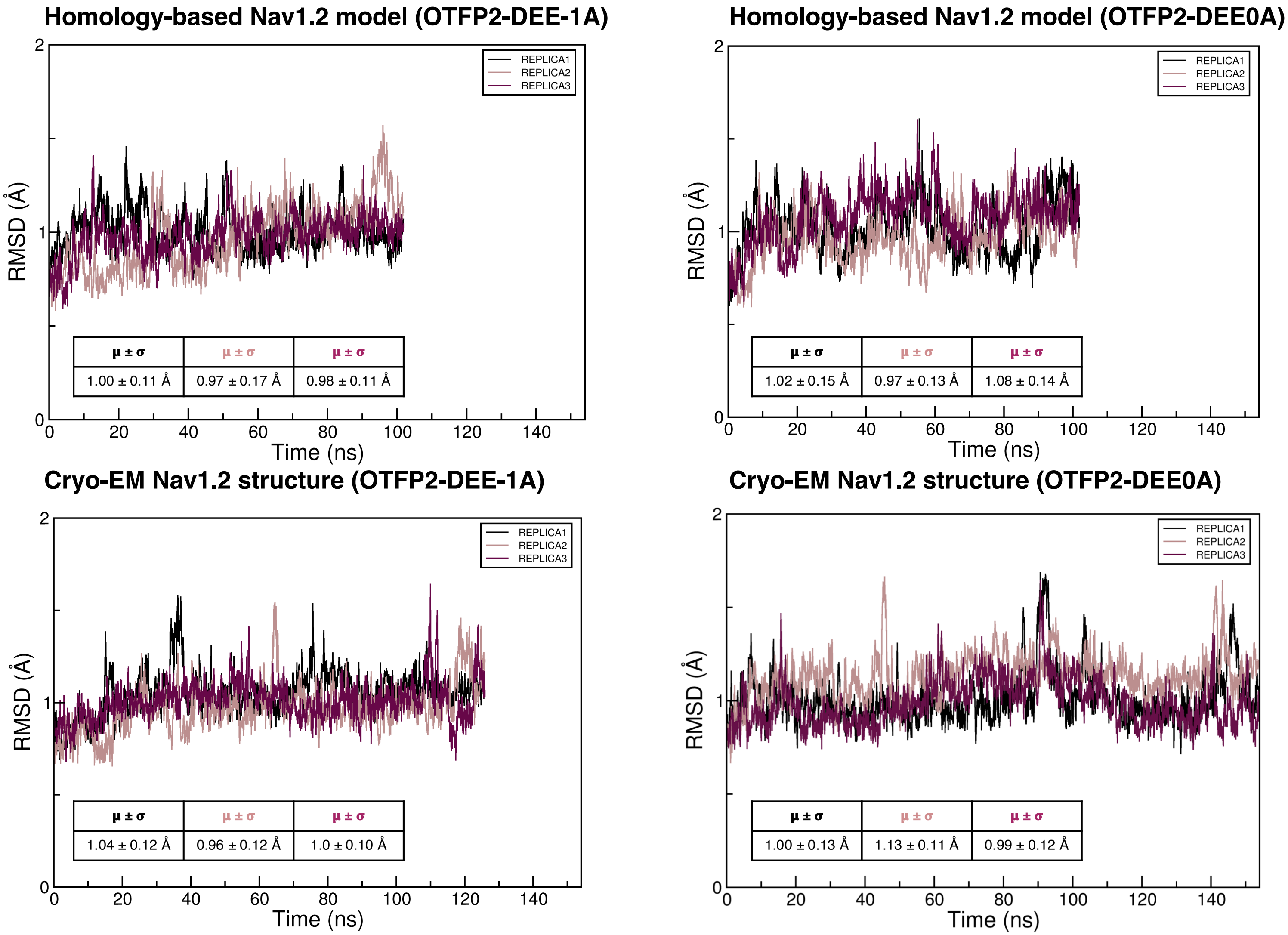

Figure S14: RMSD of the backbone P-loops (DEEA SF) for each system during the $OTFP_2$ simulations. We also report the average (μ) and the standard deviation (σ) of the RMSD. For each calculation, we report the average (μ and the standard deviation σ)

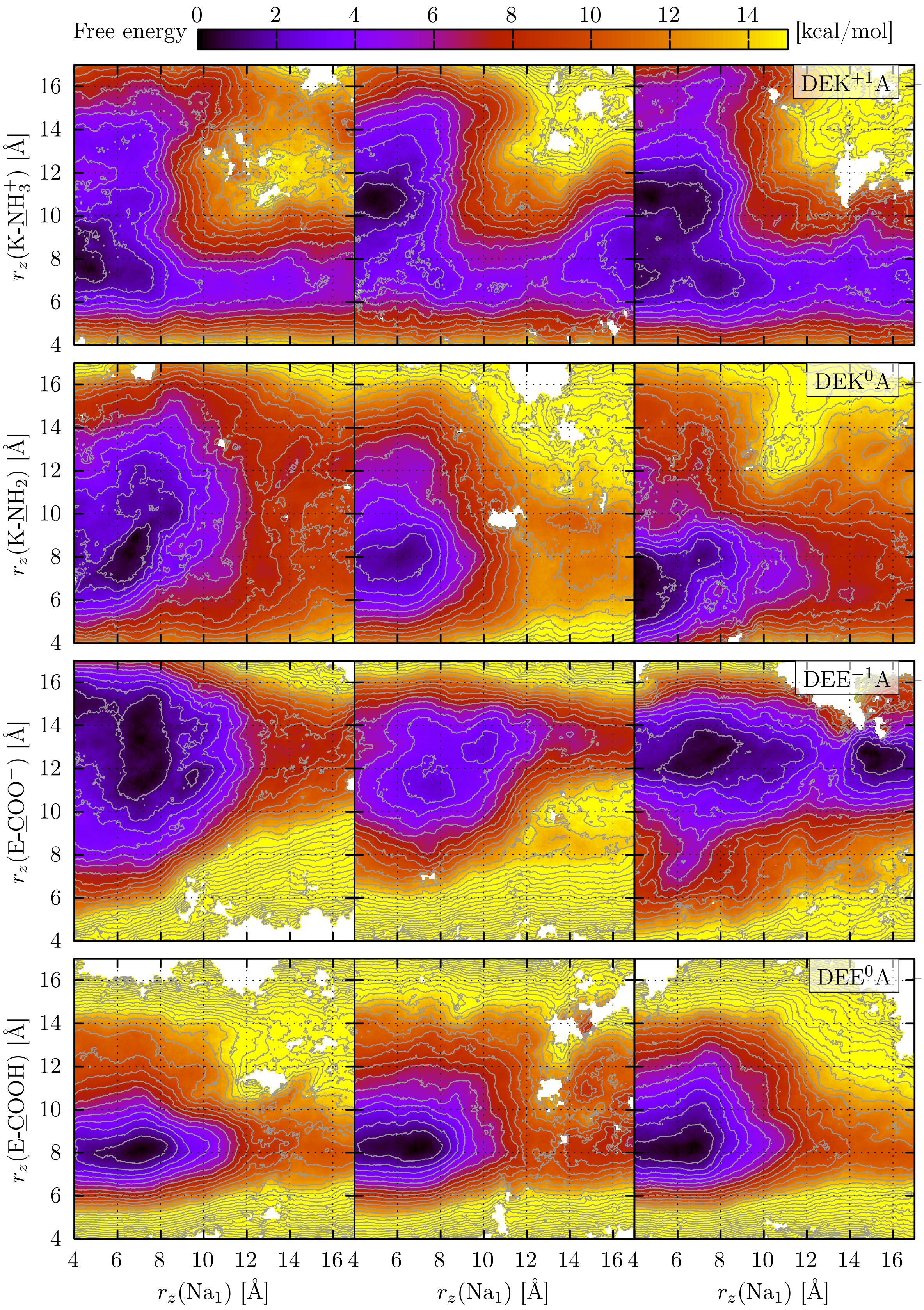

Figure S15: OTFP$_1$ FESs for single Na$^+$ translocation for each of the four systems of the homology-based Na$_\mathsf{v}$1.2 model, as indicated in the labels.

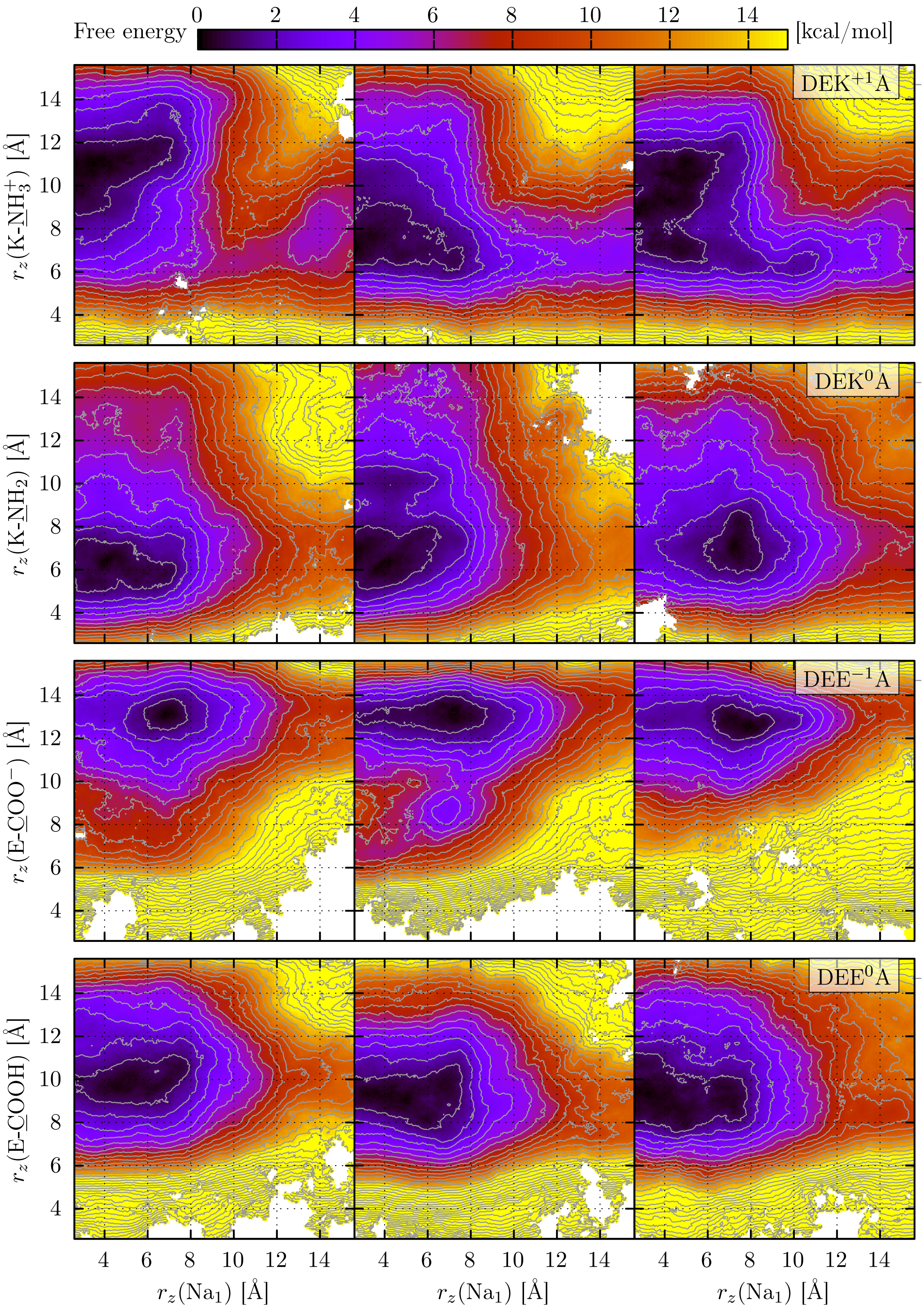

Figure S16: $OTFP_1$ FESs for single Na^+ translocation for each of the four systems of the Cryo-EM $Na_v1.2$ structure, as indicated in the labels.

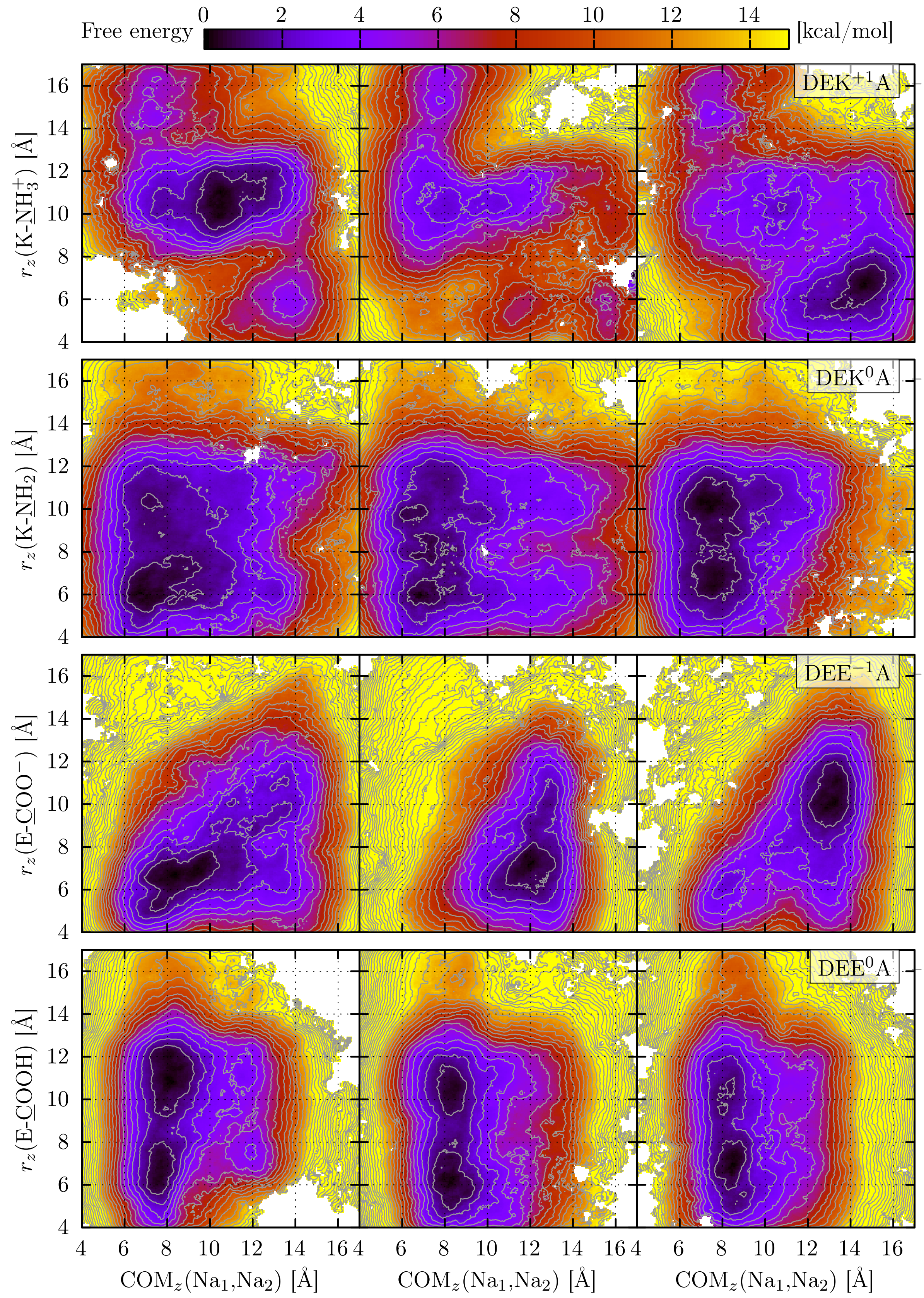

Figure S17: OTFP$_2$ FESs for double Na$^+$ translocation for each of the four systems of the homology-based Na$_\mathsf{v}$1.2 model, as indicated in the labels.

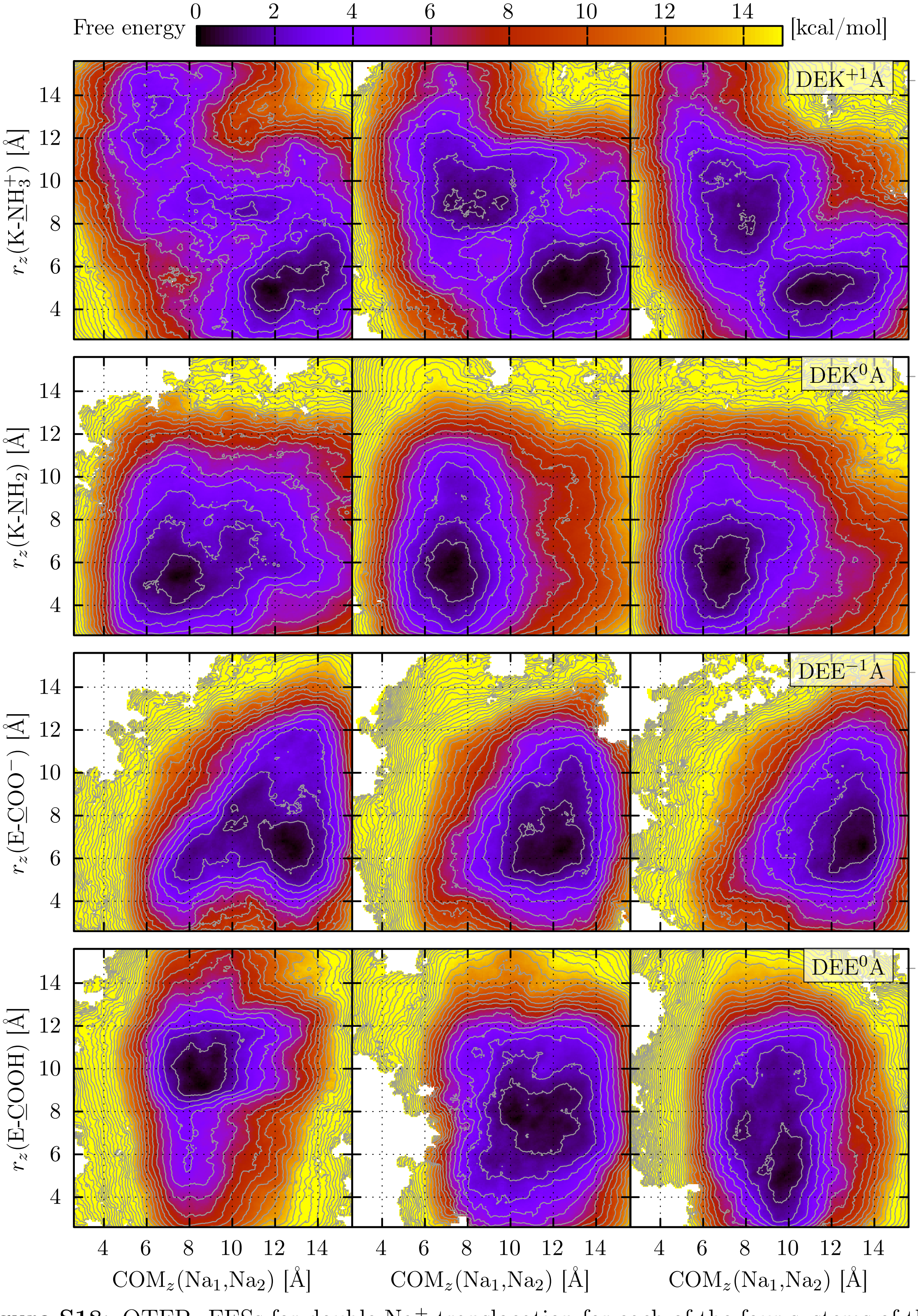

Figure S18: OTFP$_2$ FESs for double Na$^+$ translocation for each of the four systems of the Cryo-EM Na$_\mathsf{v}$1.2 structure, as indicated in the labels.

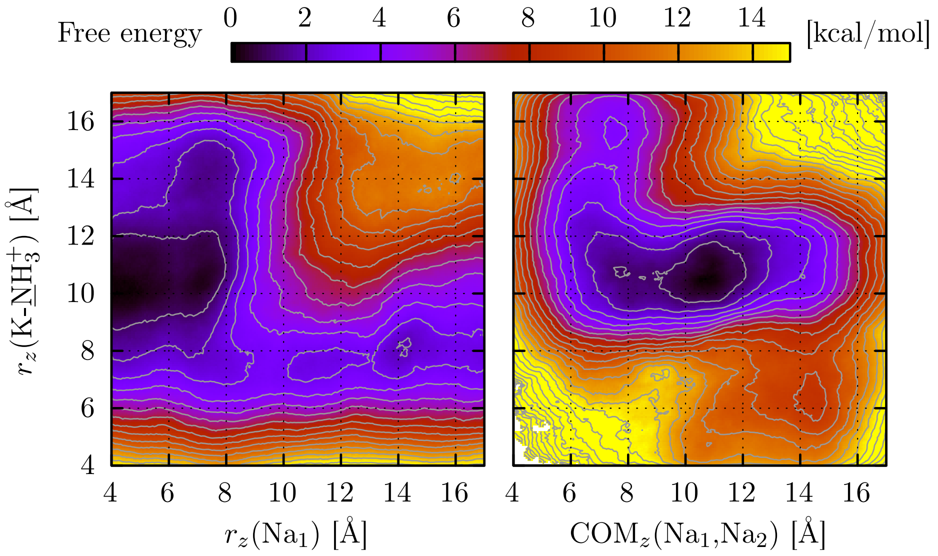

Figure S19: Average FESs based on the TAMD-OTFP simulations with a 150 mM NaCl concentration for the DEK^{+1}A system based on the homology-based Na$_v$1.2 model (OTFP$_1$: left panel, OTFP$_2$: right panel). These simulations were performed with a larger rectilinear exclusion zone having corners at (-6, -6, -4) and (6, 6, 22) Å.

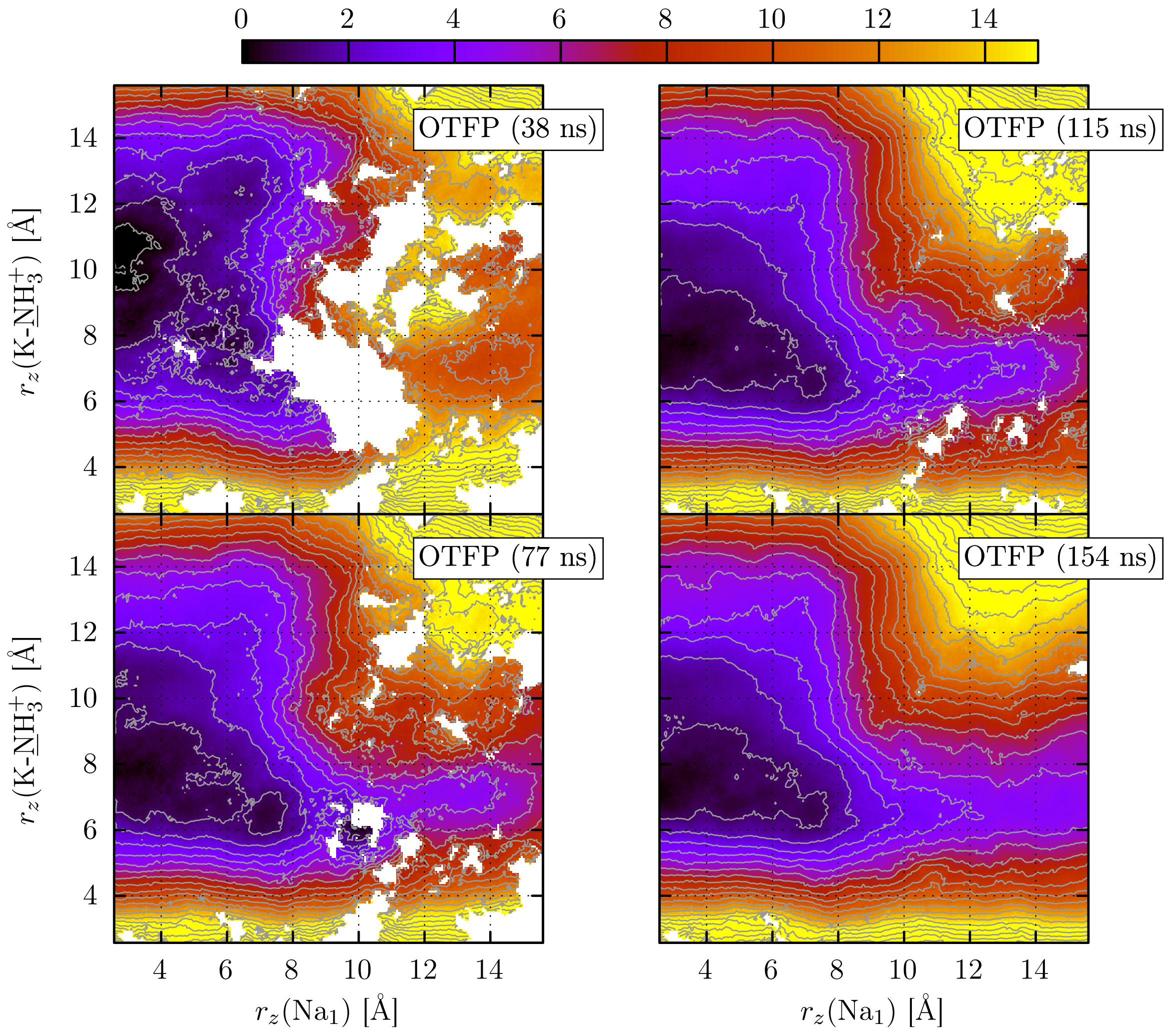

Figure S20: Example of convergence of a $OTFP_1$ FES for the $DEK^{+1}A$ system, obtained at different instances during a single simulation.

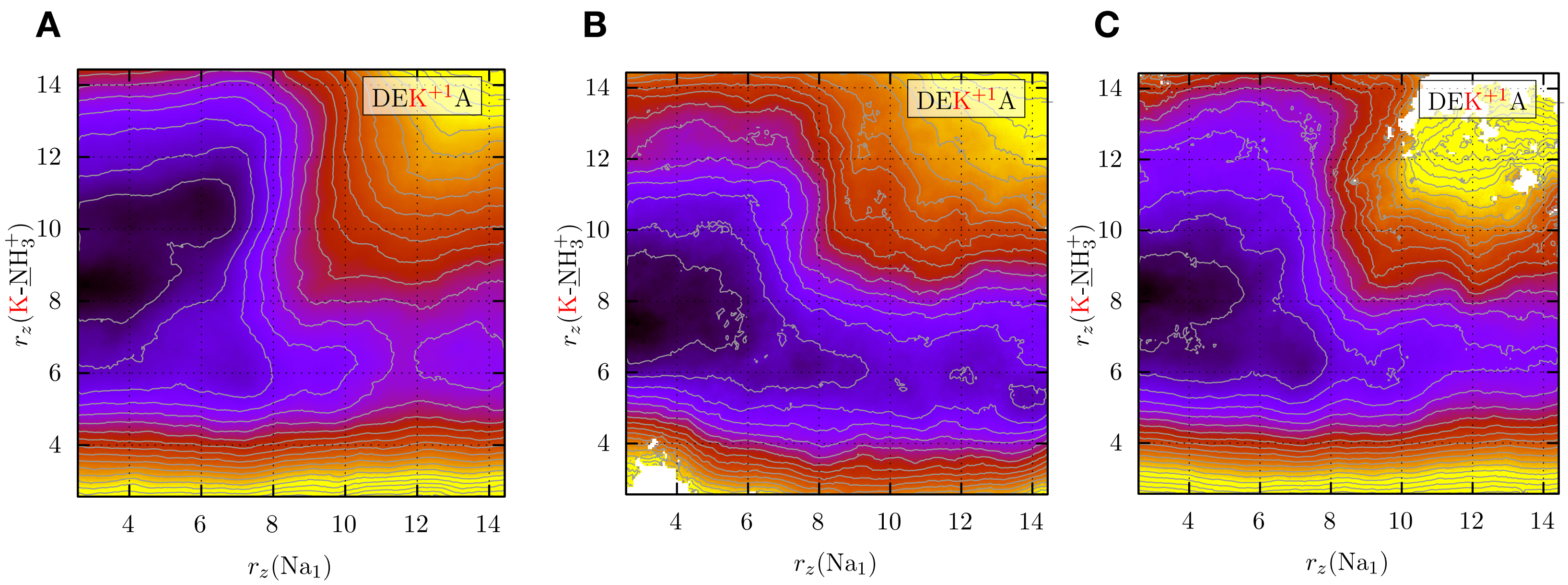

Figure S21: FESs of the OTFP$_1$ simulations for the Cryo-EM Na$_v$1.2 structure (DEK^{+1}A SF), in the case of HMR. **A** Averaged FES from HMR REPLICA1-REPLICA3. **B** FES from HMR REPLICA2. **C** FES from HMR REPLICA3.

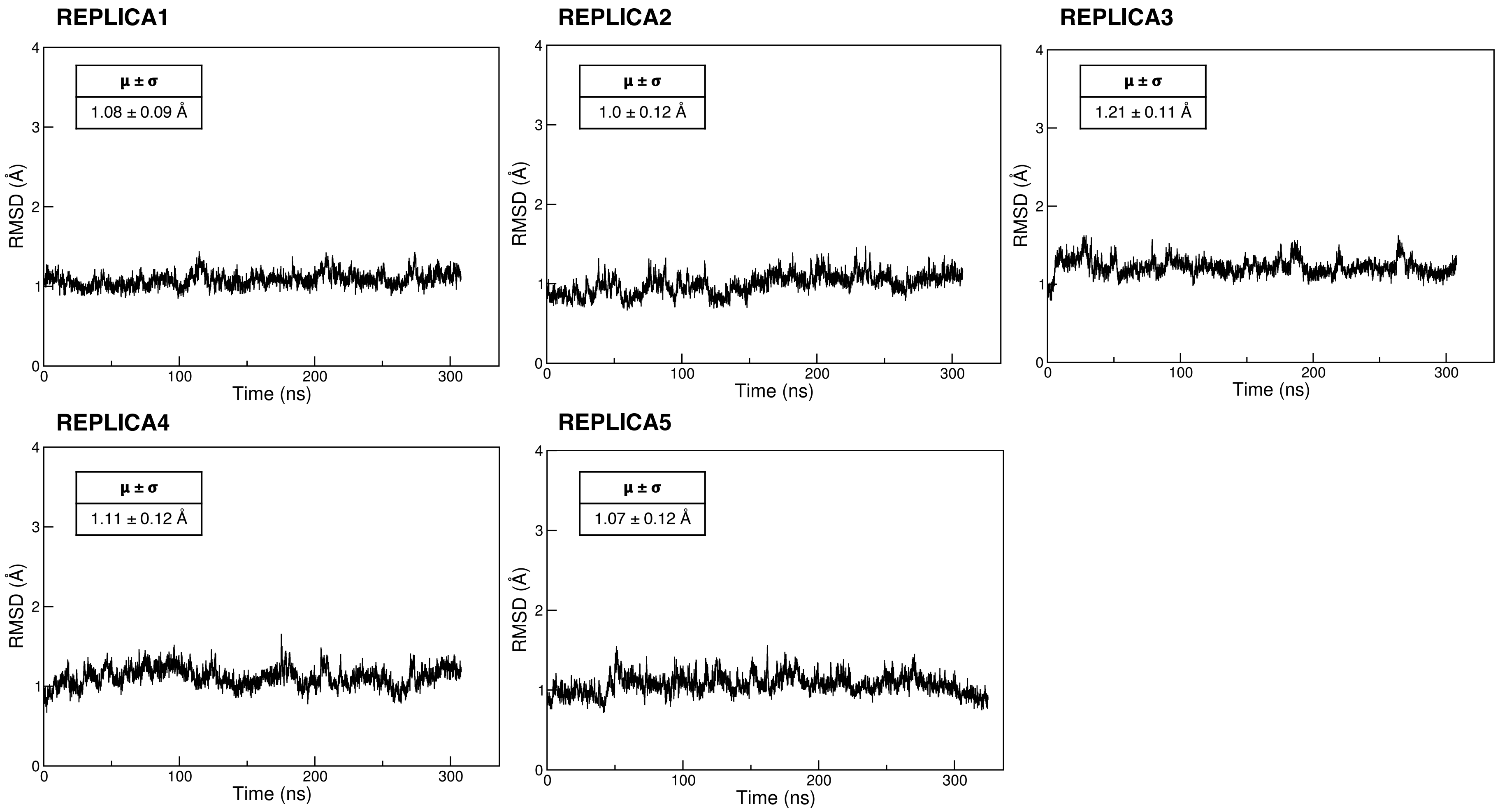

Figure S22: RMSD of the backbone P-loops (DEK^{+1}A SF) for each replica during the OTFP$_1$ simulations with HMR.

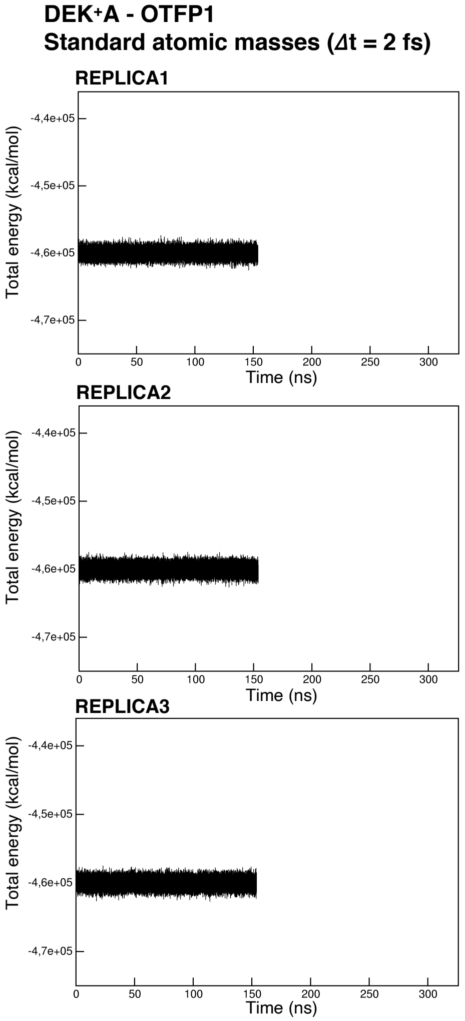

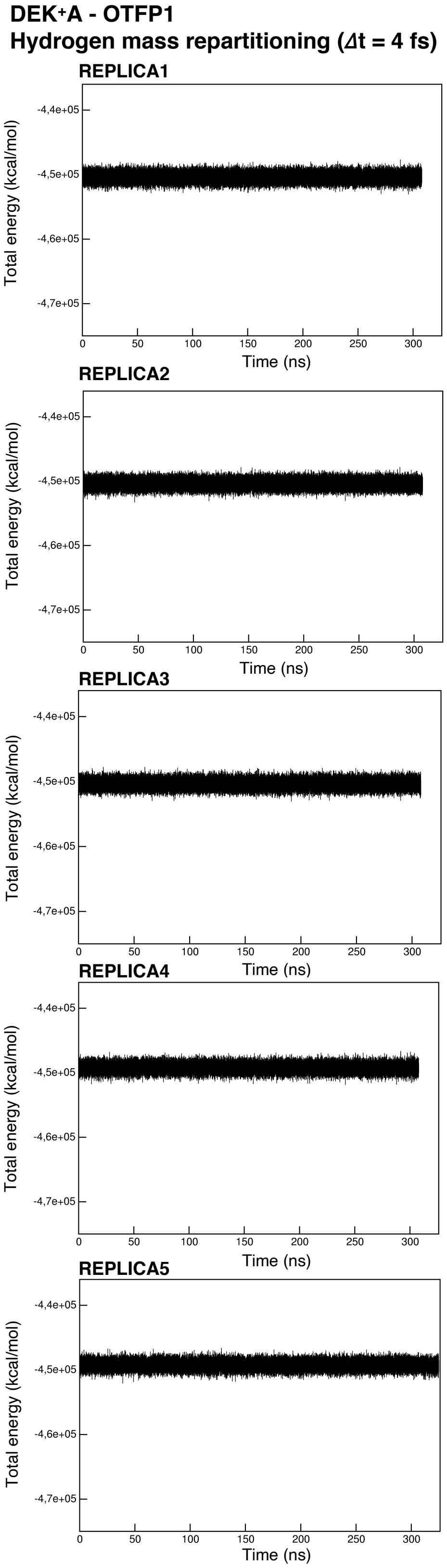

	DEK⁺A - OTFP1 Standard atomic masses (Δt= 2 fs) μ ± σ
REPLICA1	-460011 ± 560.77 kcal/mol
REPLICA2	-460025 ± 559.47 kcal/mol
REPLICA3	-460033 ± 565.59 kcal/mol

	DEK⁺A - OTFP1 Hydrogen mass repartitioning (Δt= 4 fs) μ ± σ
REPLICA1	-450399 ± 575.11 kcal/mol
REPLICA2	-450412 ± 571.18 kcal/mol
REPLICA3	-450391 ± 573.95 kcal/mol
REPLICA4	-449257 ± 577.22 kcal/mol
REPLICA5	-449265 ± 577.14 kcal mol

Figure S23: Total energy during $OTFP_1$ simulations for the Cryo-EM $Na_v1.2$ structure ($DEK^{+1}A$ SF) for the set-up with standard atomic masses with $\Delta t = 2$ fs (Replica1-3, on the left) and for HMR with $\Delta t = 4$ fs (Replica1-5, on the right). We also report the average μ and the standard deviation σ for each MD run.

List of Movies

Single ion permeation events observed for the various WT systems. In all the movies we represent the first three repeats, following the legend of **Figure 1**: domain DI, gray; domain DII, yellow; domain DIII, green. The fourth domain is excluded for clarity. The side-chains of the most external residues belonging to the EEDD motif are represented as red sticks. The charged residues of the three represented repeats belonging to the DEKA motif are also included (D and E are represented again with red side chains. The side-chain of the lysine is shown in blue sticks, with the NZ atom shown as a sphere). The internal carbonyl oxygen atoms of the two proceeding residues in each repeat are also included as red points. The permeating Na^+ ion is represented as an orange VdW sphere. Movies are available free of charge at `https://github.com/BeatriceCorradi/Nav_Channels_movies.git`

- **Movie S1**. Permeation of a single Na^+ ion in the MD simulation of the homology-based Na_v 1.2 model (time-step $\Delta t = 2$ fs). Unrestrained production.
- **Movie S2**. Single Na^+ ion residing in the region of the internal carbonyls during the MD simulation of the homology-based Na_v 1.1 model (time-step $\Delta t = 4$ fs). Restrained equilibration.
- **Movie S3**. Permeation of a single Na^+ ion in the MD simulation of the homology-based Na_v1.2 model (time-step $\Delta t = 4$ fs). Restrained equilibration.
- **Movie S4**. Permeation of a single Na^+ ion in the MD simulation of the homology-based Na_v1.6 model (time-step $\Delta t = 4$ fs). Restrained equilibration.
- **Movie S5**. Permeation of a single Na^+ ion in the MD simulation of the Cryo-EM Na_v1.2 structure (time-step $\Delta t = 4$ fs - run 1). Unrestrained production.
- **Movie S6**. Permeation of a single Na^+ ion in the MD simulation of the Cryo-EM Na_v1.2 structure (time-step $\Delta t = 4$ fs - run 2). Restrained equilibration + unrestrained production.
